# Vibration Therapy for Poststroke Clinical Balance: A Domain-Specific Systematic Review and Multilevel Meta-Analysis

**DOI:** 10.3390/healthcare14193307

**Published:** 2026-10-03

**Authors:** Jeong-Woo Seo, Ji-Woo Seok, Jung-Dae Kim

**Affiliations:** Digital Health Research Division, Korea Institute of Oriental Medicine, Daejeon 34054, Republic of Korea; jwseo02@kiom.re.kr

**Keywords:** focal muscle vibration, meta-regression, multilevel meta-analysis, postural balance, rehabilitation, stroke, whole-body vibration

## Abstract

**Highlights:**

**What are the main findings?**

- Vibration therapy was associated with a statistically significant average improvement in poststroke clinical balance, and the effect remained significant in the randomized-trial-only analysis; however, substantial heterogeneity and prediction intervals crossing the null indicate that effects vary across clinical settings.
- Excluding studies at high or serious risk of bias attenuated the pooled effect to non-significance, and none of the seven prespecified moderators remained statistically significant after false-discovery-rate correction.

**What are the implications of the main findings?**

- Vibration therapy may be considered a potential adjunct to poststroke balance rehabilitation, but the current evidence does not support recommending a specific duration, frequency, amplitude, vibration type, frequency pattern, or stroke-stage-specific protocol.
- Future well-designed dose-ranging randomized trials should use standardized mechanical-dose reporting, clinically interpretable balance outcomes, and longer-term follow-up to determine which patients and protocols are most likely to benefit.

**Abstract:**

Background/Objectives: Balance impairment is a major target of poststroke rehabilitation, but the effectiveness of mechanical vibration therapy remains uncertain. This systematic review and multilevel meta-analysis evaluated vibration therapy for validated clinical balance outcomes after stroke and explored potential effect modifiers. The review was prospectively registered in PROSPERO (CRD420251110102). Methods: PubMed, MEDLINE, Embase, the Cochrane Library, and Web of Science were searched from inception to 31 March 2026. Nineteen controlled studies contributed 22 effect-size estimates. The primary analysis used standardized between-group changes from baseline and a three-level random-effects model with study-clustered CR2 inference. Sensitivity analyses, FDR-adjusted moderator analyses, risk-of-bias assessment, and GRADE were performed. Results: Vibration therapy improved clinical balance on average (Hedges’ g = 0.593, 95% CI 0.191–0.995; *p* = 0.006), although the 95% prediction interval crossed the null. Restriction to 16 randomized trials yielded a similar effect (g = 0.507, 95% CI 0.141–0.873; *p* = 0.010). Excluding studies at high or serious risk of bias attenuated the effect to g = 0.161 (95% CI −0.116–0.439; *p* = 0.217). None of seven moderators remained significant after FDR correction. The BBS-specific mean difference was 3.326 points (95% CI 1.714–4.938). Certainty was low for randomized and very low for non-randomized evidence. Conclusions: Vibration therapy may improve poststroke clinical balance on average, but heterogeneity and sensitivity to study quality limit confidence in effect magnitude and reproducibility. Current evidence does not support a specific vibration protocol; rigorous dose-ranging randomized trials are needed.

## 1. Introduction

Stroke remains a leading cause of long-term adult disability and imposes sustained personal, social, and healthcare burdens worldwide [1]. Balance dysfunction is among the most common and consequential poststroke impairments, affecting approximately 83% of individuals with stroke in clinical cohorts [2]. Hemiparesis, muscle weakness, somatosensory loss, abnormal muscle tone, impaired anticipatory and reactive postural adjustments, and altered sensory integration collectively compromise the ability to maintain and recover stability. These deficits restrict transfers, standing, and walking; reduce independence in activities of daily living; and increase fall risk. Falls have been reported in a substantial proportion of stroke survivors after discharge and may lead to injury, fear of falling, activity restriction, and further loss of mobility [3,4]. Restoration of balance is therefore a central objective of stroke rehabilitation.

Vibration therapy (VT) has increasingly been investigated as a non-invasive adjunct to conventional rehabilitation. VT includes whole-body vibration (WBV), in which oscillatory mechanical energy is transmitted through a platform, and localized approaches applied to muscles, tendons, or the plantar surface. Whole-body vibration distributes oscillatory energy through the support surface to multiple body segments, whereas focal or localized vibration delivers mechanical stimulation directly to a selected muscle, tendon, or plantar region and therefore provides more spatially targeted afferent input. Mechanical vibration activates muscle-spindle group Ia afferents and may evoke tonic vibration reflexes, facilitate motor-unit recruitment, and alter proprioceptive input [5,6]. Repeated focal vibration has also been associated with changes in corticospinal excitability and sensorimotor cortical organization after stroke [7,8]. These peripheral and central responses provide a plausible mechanism through which VT could improve postural muscle activation, weight shifting, lower-limb proprioception, and sensorimotor integration.

Despite this biological rationale, the clinical evidence remains inconsistent. Earlier reviews of chronic stroke found little or no improvement in Berg Balance Scale scores or mobility after WBV [9,10], whereas later meta-analyses reported small to substantial benefits for balance or fall-risk factors [11,12,13,14]. Individual trials have been similarly discordant: some studies have reported improved balance after repeated vibration, while rigorous sham-controlled trials have found no specific effect [15,16]. Such disagreement suggests that VT should not be treated as a single uniform intervention.

Vibration protocols differ in modality, frequency, amplitude, oscillation direction, body position, exercise task, session duration, weekly frequency, total treatment duration, and co-intervention. Patient characteristics, particularly stroke stage and baseline impairment, may further modify treatment response. In addition, balance is multidimensional. Static postural control, dynamic or composite balance, limits of stability, functional mobility, and gait performance represent related but non-equivalent constructs. Pooling these outcomes solely because they are described as balance-related can obscure domain-specific effects and produce clinically ambiguous summary estimates.

To address these limitations, we prospectively registered and published a domain-specific protocol that specified construct-based outcome classification, separate synthesis of conceptually comparable outcomes, and exploratory analysis of vibration parameters [17]. The protocol emphasized that standardized mean differences should not be used to justify pooling measures with different underlying constructs or task demands. The present report focuses on validated clinical scales of dynamic or composite balance, the domain for which a sufficiently coherent controlled evidence base was available. Instrumented posturography, functional mobility, and gait-speed outcomes were not pooled with clinical balance scales.

The primary objective of this systematic review and multilevel meta-analysis was to quantify the effect of VT on clinical balance function in adults after stroke. Secondary objectives were to determine whether vibration type, frequency, frequency pattern, amplitude, weekly treatment frequency, total intervention duration, or stroke stage explained heterogeneity; to test whether the result was robust when restricted to randomized trials; and to evaluate the certainty of the evidence using GRADE. By integrating construct-specific synthesis, dependency-aware meta-analysis, moderator assessment, and evidence-certainty appraisal, this study sought to provide a more precise basis for clinical interpretation and future protocol development.

## 2. Materials and Methods

### 2.1. Protocol, Registration, and Reporting

This systematic review and meta-analysis was conducted in accordance with PRISMA 2020 [18] and represents the completed review arising from our prospectively registered protocol. The protocol was registered in PROSPERO (CRD420251110102) and subsequently published as a peer-reviewed protocol in BMJ Open [17]. Deviations from the published protocol are described explicitly below. Ethical approval was not required because the study used aggregate data from previously published reports.

The published protocol proposed separate analyses across five balance-related constructs: static postural control, dynamic or composite balance, limits of stability, functional mobility, and gait speed or performance. Consistent with the protocol principle that outcomes should be pooled only when they measure a conceptually comparable construct, the present quantitative report focused on validated clinical measures of dynamic or composite balance. The primary synthesis included the Berg Balance Scale, Mini-Balance Evaluation Systems Test, and the balance or total components of the Tinetti Performance-Oriented Mobility Assessment. Timed Up and Go, gait-speed, Functional Reach Test, instrumented posturography, balance-confidence, trunk-control, and ambulation outcomes were tabulated but were not combined with the primary clinical balance construct. Multiple eligible intervention arms or outcomes from the same parent study were retained as separate effect-size estimates. Dependence was addressed using a three-level random-effects structure together with cluster-robust variance estimation with a CR2 small-sample correction at the study level.

#### Deviations from the Published Protocol

All deviations from the published protocol were documented transparently. First, the completed database search used PubMed, MEDLINE, Embase, the Cochrane Library, and Web of Science; Scopus, which had been listed in the protocol, was not searched, whereas MEDLINE was searched separately. Second, although the protocol proposed separate syntheses across five balance-related constructs, quantitative synthesis in the present report was restricted to validated clinical measures of dynamic or composite balance because this was the only construct with a sufficiently coherent controlled evidence base for pooling. Other balance-related outcomes were retained descriptively.

Third, the protocol allowed flexibility in effect-size selection when reporting was insufficient. In the final analysis, we standardized the primary effect-size definition to the between-group difference in change from baseline rather than mixing change-score and post-intervention estimates. This refinement was made to address baseline imbalance consistently across randomized and non-randomized studies. Details of the effect-size hierarchy and assumed pre–post correlations are provided below.

Fourth, because multiple eligible estimates arose from several studies, the primary synthesis used a three-level random-effects model supplemented by study-cluster robust variance estimation with the CR2 small-sample correction. Additional sensitivity analyses examined assumptions regarding pre–post correlations and within-study dependence.

Fifth, moderator analyses were restricted to variables that could be coded consistently across studies. Session duration, total intervention volume, and multivariable meta-regression were not performed because reporting was insufficient and the number of studies was inadequate for stable multivariable estimation. Because seven moderators were evaluated, false-discovery-rate correction was applied to address multiplicity.

Finally, the protocol-planned robustness analyses included exclusion of studies at high or serious risk of bias, leave-one-study-out analyses, influence and outlier diagnostics, restriction to the same balance instrument, exclusion of the TPOMA total score, exclusion of the study with identified baseline imbalance, and restriction to randomized trials.

### 2.2. Eligibility Criteria

Studies were eligible if they included adults aged 18 years or older with a clinical diagnosis of ischemic or hemorrhagic stroke, regardless of whether participants were in the acute, subacute, postacute, or chronic stage. Studies involving pediatric populations or non-stroke neurological disorders were excluded unless stroke-specific data were separately extractable.

Eligible interventions delivered mechanical vibration to the whole body or to a localized muscle, tendon, or plantar region. WBV, focal muscle vibration, local muscle vibration, plantar vibration, and proprioceptive body vibration were eligible when stimulation parameters and the intervention schedule were sufficiently described. Studies combining VT with another rehabilitation intervention were included only when the comparator received the same co-intervention without therapeutic vibration, thereby allowing the incremental effect of vibration to be estimated. Manual vibration, auditory cueing, acoustic or ultrasound treatment, and vibration used solely as an external sensory cue were excluded.

Acceptable comparators included sham or placebo vibration, conventional rehabilitation, active exercise or rehabilitation controls, usual care, or an equivalent co-intervention without vibration. Randomized controlled trials, non-randomized controlled trials, and controlled crossover studies with extractable quantitative data were eligible. Case reports, case series, uncontrolled pre-post studies, cross-sectional studies, reviews, editorials, and conference abstracts without full data were excluded.

For this domain-specific analysis, studies had to report an extractable validated clinical measure of dynamic or composite balance. Clinical dynamic/composite balance was operationally defined as performance on a validated multi-item clinical scale that integrates several components of postural control, such as transfers, standing balance, anticipatory control, dynamic stability, or gait-related balance, into an overall balance-related score. Eligible primary outcomes were the Berg Balance Scale (BBS), Mini-Balance Evaluation Systems Test (Mini-BESTest), and the balance or total components of the Tinetti Performance-Oriented Mobility Assessment (TPOMA). TPOMA total scores were retained only when the reported score represented the instrument’s combined balance and gait-related postural performance and was judged clinically comparable with the composite balance construct. Timed Up and Go, gait speed, Functional Reach Test, instrumented posturography, balance-confidence, trunk-control, and ambulation outcomes were retained in the descriptive study table but excluded from the primary quantitative synthesis because they represent distinct task demands or constructs.

### 2.3. Information Sources and Search Strategy

PubMed, MEDLINE, Embase, the Cochrane Library, and Web of Science were searched from database inception to 31 March 2026. Search strings combined controlled vocabulary and free-text terms for stroke, mechanical vibration, and balance or postural control. Representative terms included stroke, cerebrovascular accident, hemiplegia, vibration therapy, whole-body vibration, focal muscle vibration, balance, postural control, and postural stability. The completed search record therefore differed from the protocol database list, which had included Scopus; this deviation is reported transparently. The prespecified database search strategy is available in the published protocol and its online supplementary appendix [17]. The complete database-specific search strategies actually used in the final review are provided in Supplementary Table S1.

Backward citation searching of included studies and relevant reviews and forward citation tracking were performed to identify additional eligible reports. No language restriction was applied at the search stage. At full-text review, one non-English report was excluded because the quantitative data required for reliable effect-size extraction could not be obtained; the reason was therefore data availability rather than language alone.

### 2.4. Study Selection

All records were imported into reference-management software and duplicates were removed. Two reviewers independently screened titles and abstracts, retrieved potentially eligible full texts, and assessed full-text reports against the eligibility criteria. Disagreements were resolved by discussion and consensus; a third reviewer adjudicated unresolved decisions. The study-selection process was documented using a PRISMA 2020 flow diagram [18].

### 2.5. Data Extraction and Outcome Coding

Two reviewers independently extracted data using a standardized, pilot-tested form. Extracted study characteristics included author, publication year, design, sample size, age, stroke stage, intervention and comparator details, device, stimulation site, posture, co-interventions, treatment frequency, total duration, and all reported balance-related outcomes. The outcomes contributing to the primary meta-analysis were coded separately from additional functional mobility, gait, posturographic, confidence, trunk-control, and ambulation measures. Discrepancies were resolved by consensus with third-reviewer consultation when required.

Intervention parameters were coded using the Body Vibration Big Five framework: vibration frequency, amplitude, application method, session frequency, and total intervention duration [6]. Frequency pattern and stroke stage were additionally coded as candidate moderators. For studies with multiple eligible intervention arms or eligible clinical balance outcomes, each eligible comparison was extracted separately and linked to its parent study. The 19 included studies therefore contributed 22 effect-size estimates: multiple estimates arose from multiple eligible intervention arms, multiple eligible outcomes, or both. Specifically, effect sizes originating from the same parent study were assigned a common study identifier and retained within the multilevel structure. Because complete arm-level information required to reconstruct the exact sampling covariance associated with shared comparator groups was not consistently available across reports, a complete sampling variance-covariance matrix could not be reconstructed for all studies. Accordingly, the primary inferential analysis combined the multilevel random-effects structure with study-clustered CR2 robust variance estimation. Sensitivity analyses additionally examined plausible assumptions regarding within-study dependence.

For the primary meta-analysis, we extracted pre-intervention and immediate post-intervention means, standard deviations, and sample sizes for the intervention and control groups, together with directly reported change scores and change-score standard deviations when available. The primary effect estimate was defined as the standardized between-group difference in change from baseline. Directly reported change means and standard deviations were used preferentially. When change means were not reported, they were calculated as the post-intervention mean minus the baseline mean. When the standard deviation of the change score was unavailable, it was reconstructed from the pre- and post-intervention standard deviations using the Cochrane variance formula and an assumed within-group pre-post correlation of r = 0.50. Sensitivity analyses repeated the effect-size calculations under alternative correlation assumptions. Hedges’ small-sample correction was subsequently applied to all standardized mean differences [19]. The effect-size-level extraction dataset, including sample sizes, change scores, reconstructed change-score standard deviations, Hedges’ g values, and sampling variances, is provided in Supplementary Table S6.

The immediate post-treatment assessment was prespecified as the primary time point for evaluating short-term treatment effects. Follow-up measurements were extracted separately when reported to characterize durability but were not combined with immediate post-treatment estimates in the primary synthesis. Outcome directions were harmonized so that positive effect sizes represented greater improvement in balance with vibration therapy.

### 2.6. Risk of Bias Assessment

Two reviewers independently assessed randomized trials using the Cochrane Risk of Bias 2 tool across the randomization process, deviations from intended interventions, missing outcome data, outcome measurement, and selection of the reported result [20]. Non-randomized studies were assessed using ROBINS-I across confounding, participant selection, intervention classification, deviations from intended interventions, missing data, outcome measurement, and selective reporting [21]. Disagreements were resolved through consensus or third-reviewer adjudication.

### 2.7. Statistical Analysis

All statistical analyses were performed in R version 4.5.2 using the metafor package [22] and the clubSandwich package. The primary effect measure was the standardized between-group difference in change from baseline, expressed as Hedges’ *g* with correction for small-sample bias according to established meta-analytic procedures [19]. Positive values indicated greater improvement in balance with vibration therapy. Change-score means and standard deviations were obtained as described above, with *r* = 0.50 used as the primary pre-post correlation when change-score standard deviations required reconstruction.

Because some studies contributed multiple eligible intervention arms or outcomes, the primary synthesis used a three-level random-effects model estimated by restricted maximum likelihood (REML), with effect-size estimates nested within parent studies [23,24]. Sampling variance constituted level 1, residual within-study variance level 2, and between-study variance level 3. Importantly, the multilevel structure was not assumed to fully resolve sampling dependence arising from shared participants or comparator groups. Statistical inference for the pooled effect was therefore based on cluster-robust variance estimation with the CR2 small-sample correction at the parent-study level and Satterthwaite-adjusted degrees of freedom.

Residual heterogeneity was evaluated using the *Q*E statistic, level-specific variance components (τ^2^ and τ), and 95% prediction intervals. Variance-component confidence intervals and model-fit diagnostics were additionally examined using profile-likelihood and variance-component comparisons where applicable.

Prespecified univariable multilevel meta-regression analyses evaluated vibration type, vibration frequency, frequency pattern, weekly treatment frequency, total intervention duration, amplitude, and stroke stage. Each moderator was analyzed using the same multilevel REML framework with study-clustered CR2 inference [23,24]. The number of contributing parent studies and effect-size estimates was reported for each moderator category. Residual heterogeneity and changes in between-study variance (τ^2^) after moderator inclusion were examined as indicators of explained heterogeneity. Because multiple moderators were evaluated in a relatively small evidence base, Benjamini–Hochberg false discovery rate (FDR) correction was applied across the seven omnibus moderator tests; both unadjusted and FDR-adjusted *p* values are reported. These analyses were considered exploratory and hypothesis-generating.

Exploratory subgroup analyses were conducted for the same clinical and intervention variables. Within each subgroup containing at least two independent parent studies, effect sizes were synthesized using a multilevel REML model with study-clustered CR2 inference when multiple estimates were contributed by the same study; conventional random-effects models were used when all contributing estimates were independent. The numbers of independent parent studies and effect-size estimates were reported separately for each subgroup. Subgroup-specific pooled effects were used to characterize effects within categories, whereas differences between categories were evaluated using the corresponding CR2 omnibus moderator tests with FDR adjustment.

Sensitivity analyses evaluated the robustness of the primary findings to study design, risk of bias, outcome definition, measurement instrument, baseline imbalance, assessment time point, and analytic assumptions. These analyses included restriction to randomized controlled trials; exclusion of studies at high or serious risk of bias; exclusion of TPOMA total while retaining TPOMA balance; restriction to BBS outcomes; exclusion of the study with identified baseline imbalance; and restriction to the primary post-treatment assessment. The influence of assumptions used to reconstruct change-score variances was examined by recalculating effect sizes using pre-post correlations of r = 0.25 and 0.75, in addition to the primary assumption of r = 0.50. Sensitivity to within-study dependence was further examined using assumed within-study correlations of ρ = 0.30, 0.50, and 0.70. The complete results of these sensitivity and robustness analyses are summarized in Supplementary Table S7.

Leave-one-study-out analyses removed one entire parent study at a time rather than individual effect-size estimates. Study-deletion changes in the pooled effect and heterogeneity were used to assess influence. Effect-level standardized residuals were additionally examined to identify potential statistical outliers, using an absolute standardized residual >1.96 as the diagnostic threshold, followed by an analysis excluding the parent study containing an identified outlying estimate.

Potential small-study effects were assessed by funnel-plot inspection and complementary regression- and rank-based diagnostics, including an Egger-type meta-regression, weighted regression, and the Begg–Mazumdar rank-correlation test [25]. Trim-and-fill was retained as an additional exploratory diagnostic. These conventional diagnostics were conducted at the effect-size level and therefore did not fully account for within-study dependence; consequently, they were interpreted as secondary exploratory assessments rather than definitive tests of publication bias.

To improve clinical interpretability, an additional clinical-scale analysis was conducted for studies reporting the Berg Balance Scale. Raw between-group mean differences in BBS change scores were calculated in scale points and synthesized using the same multilevel REML and study-clustered CR2 framework. Positive mean differences indicated greater improvement with vibration therapy. Statistical tests were two-sided, with *p* < 0.05 considered statistically significant before multiplicity adjustment where applicable.

Study characteristics and individual-study results were summarized in tables, and meta-analytic estimates were displayed using forest plots. Leave-one-study-out results were displayed graphically, and small-study effects were examined visually using a funnel plot in addition to the statistical diagnostics described above.

### 2.8. Certainty of Evidence

The certainty of evidence was evaluated using the Grading of Recommendations Assessment, Development and Evaluation (GRADE) framework across five domains: risk of bias, inconsistency, indirectness, imprecision, and publication bias [26,27]. Randomized and non-randomized evidence were evaluated separately. Randomized evidence initially received a high-certainty rating, whereas non-randomized evidence initially received a low-certainty rating, with subsequent downgrading or upgrading as appropriate according to GRADE guidance [26,27]. Two reviewers independently assessed the certainty of evidence, and disagreements were resolved through discussion and consensus.

Downgrading decisions were made separately for each GRADE domain, and the rationale for each decision was documented explicitly in the Summary of Findings table. Risk of bias was judged using the design-specific RoB 2 and ROBINS-I assessments together with the results of sensitivity analyses excluding studies at high or serious risk of bias. Inconsistency was evaluated based on the magnitude and statistical evidence of residual heterogeneity, variation in study-level effect estimates, and the 95% prediction interval.

For imprecision, judgments were based primarily on the width of the 95% confidence interval around the pooled effect, the amount of independent evidence contributing to the estimate, and whether the confidence interval encompassed materially different interpretations of the treatment effect, including no effect, in accordance with GRADE guidance for continuous outcomes [26,27]. Prediction intervals were used to characterize inconsistency and the expected variation of effects across clinical settings rather than as the primary criterion for imprecision. Publication bias was assessed from the overall pattern of the funnel plot and complementary small-study-effect diagnostics, recognizing the limitations of conventional asymmetry tests when multiple effect-size estimates are contributed by the same study. The complete evidence profile, including the pooled standardized effect estimates with 95% confidence intervals and the explicit reason for each downgrade, is presented in Supplementary Table S5.

## 3. Results

### 3.1. Study Selection Results

**Figure 1:**
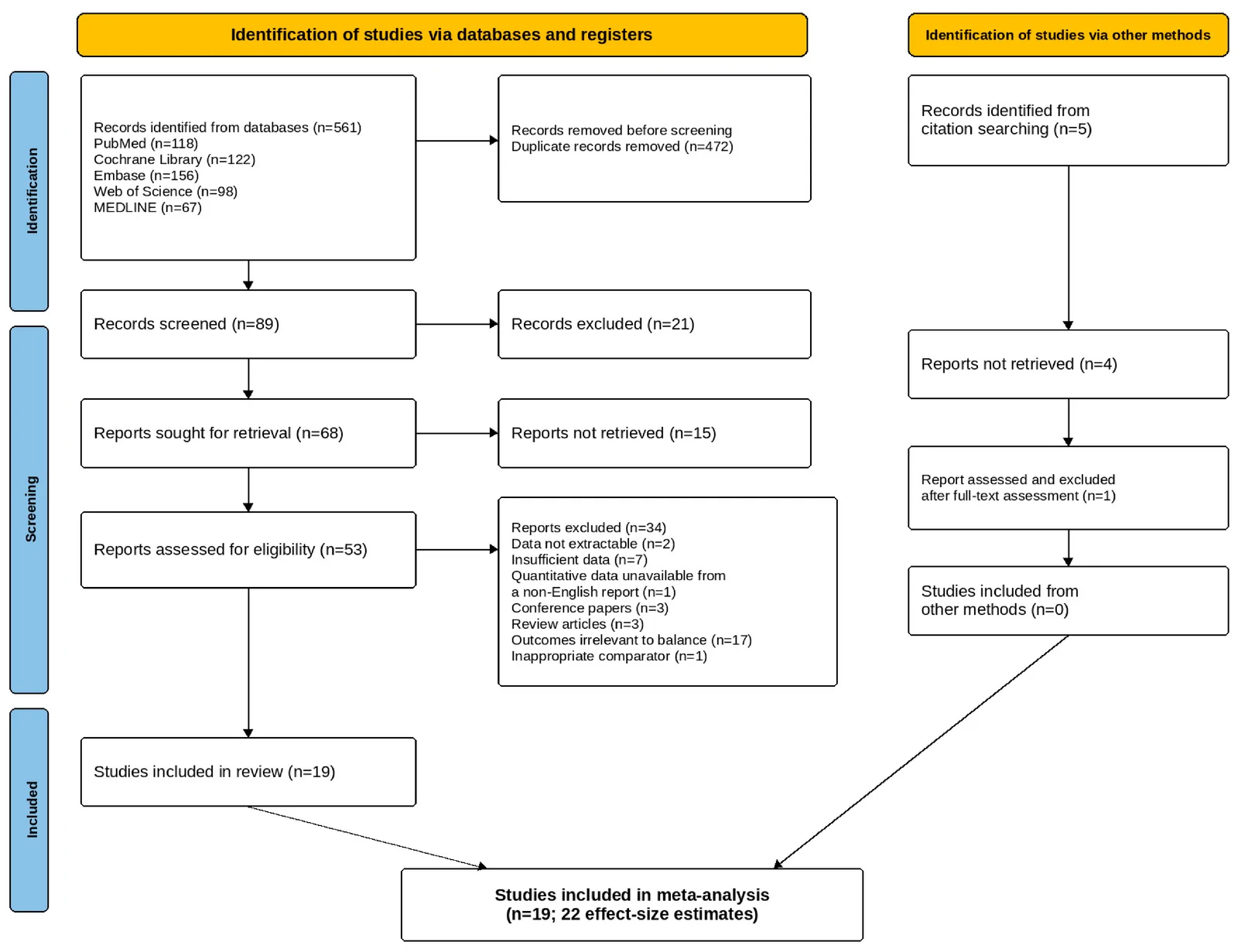
PRISMA flow diagram of the study selection process.

Database searching identified 561 records: PubMed (n = 118), Cochrane (n = 122), Embase (n = 156), Web of Science (n = 98), and MEDLINE (n = 67). Citation searching identified five additional records. After removal of 472 duplicates, 89 records were screened and 21 were excluded. Sixty-eight database reports were sought for retrieval; 15 could not be retrieved, leaving 53 reports for full-text assessment. Thirty-four full-text reports were excluded because data were not extractable (n = 2), data were insufficient (n = 7), quantitative data required for reliable extraction could not be obtained from a non-English report (n = 1), the report was a conference paper (n = 3) or review article (n = 3), outcomes were irrelevant to balance (n = 17), or the comparator was inappropriate (n = 1). Of the five citation-search records, four were not retrieved and one was assessed and excluded. Nineteen studies were included in the systematic review and meta-analysis (Figure 1).

### 3.2. Study Characteristics

The 19 included studies comprised 16 randomized controlled trials, two retrospective controlled studies, and one prospective cohort study. Thirteen studies enrolled participants with chronic stroke, three enrolled subacute participants, and three enrolled postacute participants. Total sample size per study ranged from 20 to 130 participants, and reported intervention or control group mean ages ranged from 46.8 to 74.5 years. Multi-arm studies contributed separate comparisons, and the primary analysis contained 22 comparisons from 19 studies.

WBV was the predominant intervention. The evidence base also included local muscle vibration applied to the quadriceps tendon, plantar vibration applied to the affected foot, and proprioceptive body vibration. Comparators included sham vibration, conventional physical therapy, rehabilitation, exercise, physiotherapy, and routine rehabilitation. Vibration frequency ranged from 1 to 40 Hz and reported amplitude from 0.2 to 30 mm. Treatment schedules ranged from 1–2 sessions per week to daily or six sessions per week, and total intervention duration ranged from a single session to 12 weeks.

Interventions were delivered during standing, semi-squat, horizontal standing, sitting, supine positioning, assisted squats, or weight-shifting tasks, while localized stimulation targeted the quadriceps tendon or plantar surface. Devices included Galileo systems, Gymna Fitvibe, Jet-Vibe, Power Plate, Sonix, Vibrosphere, and other vibration platforms. The included studies reported BBS, Mini-BESTest, TPOMA, TUG, Functional Reach Test, posturographic measures, balance-confidence scales, trunk-control measures, and ambulation outcomes. The primary meta-analysis used BBS, Mini-BESTest, and TPOMA balance or total outcomes; full study characteristics and all reported outcomes are presented in Table 1.

Safety, withdrawals, and adherence were inconsistently reported across the included studies. Several studies explicitly reported no intervention-related adverse events, whereas others did not report safety outcomes. Reported symptoms were generally mild and transient, including muscle soreness or fatigue, dizziness, and knee pain. Quantitative adherence measures were infrequently reported. Study-level safety, withdrawal, and adherence information is provided in Supplementary Table S2.

### 3.3. Risk of Bias

Among the 16 randomized trials, seven (43.8%) were judged to have low risk of bias, one (6.3%) raised some concerns, and eight (50.0%) had high risk of bias. The randomization process was the most frequent source of concern, particularly when sequence generation or allocation concealment was insufficiently described. Bias due to missing outcome data and selective reporting was generally low, and most studies had low risk in outcome measurement, although one trial had high risk in this domain.

Among the three non-randomized studies, two (66.7%) had serious risk of bias and one (33.3%) had moderate risk. Confounding was the principal limitation, with an additional concern regarding participant selection in one study. Across all included studies, seven (36.8%) had low risk, two (10.5%) had some concerns or moderate risk, and 10 (52.6%) had high or serious risk. Risk-of-bias summaries are presented in Supplementary Figure S1.

### 3.4. Overall Effect of Vibration Therapy on Clinical Balance

**Figure 2:**
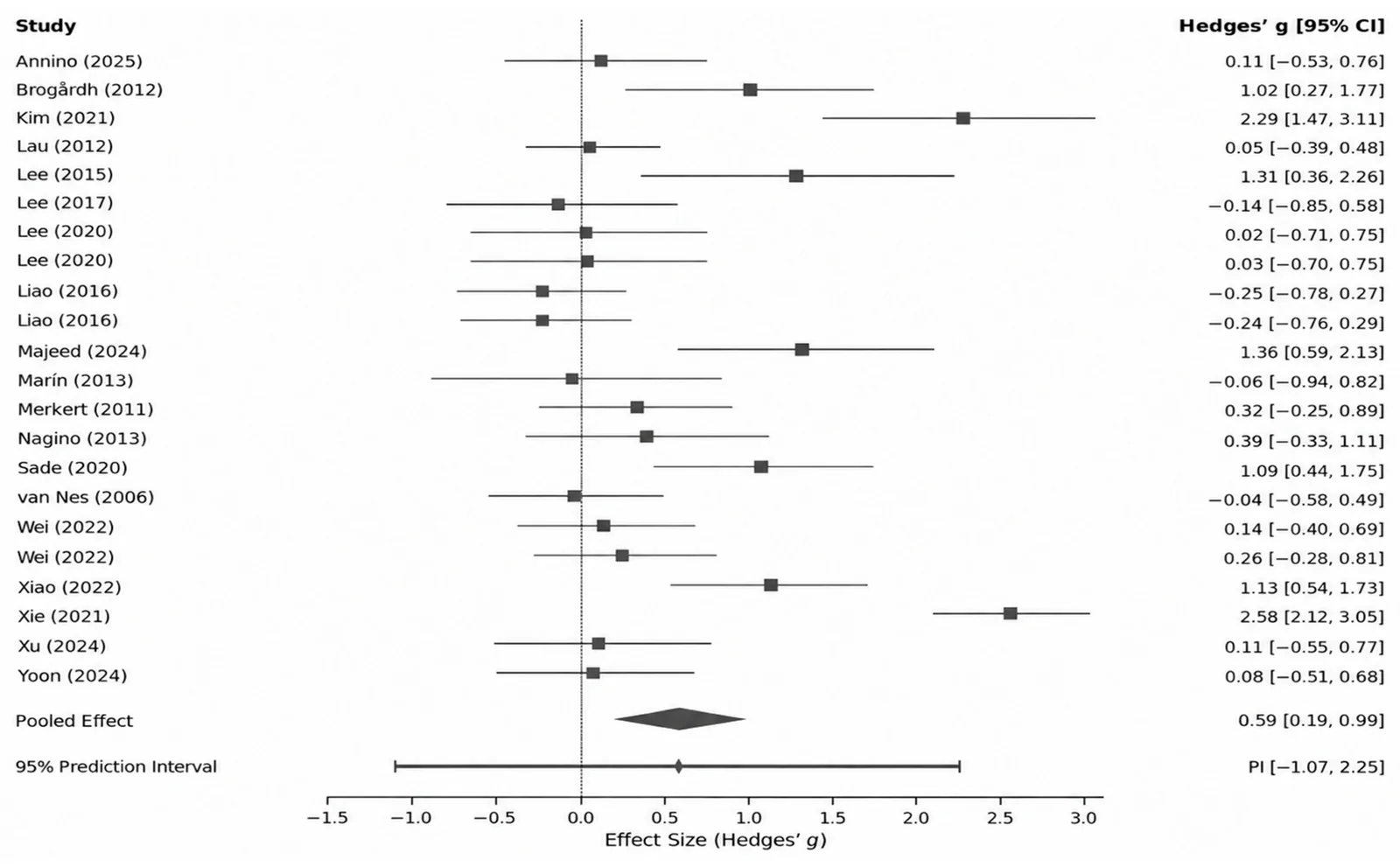
Forest plot of the multilevel meta-analysis of vibration therapy on clinical balance after stroke. The analysis included 22 effect-size estimates from 19 studies. Effect sizes represent standardized between-group differences in change from baseline (Hedges’ *g*). Squares and horizontal lines represent individual effect sizes and their 95% confidence intervals (CIs), respectively. The diamond represents the pooled estimate obtained from the three-level REML model with study-clustered CR2 inference (Hedges’ *g* = 0.593, 95% CI 0.191 to 0.995, *p* = 0.006). The lower horizontal bar represents the 95% prediction interval (−1.066 to 2.252) [15,16,28,29,30,31,32,33,34,35,36,37,38,39,40,41,42,43,44].

The primary analysis included 22 effect-size estimates from 19 studies. The three-level random-effects model with study-clustered CR2 inference showed that vibration therapy was associated with a statistically significant improvement in clinical balance compared with control interventions (Hedges’ *g* = 0.593, 95% CI 0.191 to 0.995, *p* = 0.006) (Figure 2).

Residual heterogeneity was statistically significant, *Q*E(21) = 150.98, *p* < 0.001. The estimated between-study variance was τ^2^ = 0.586 (τ = 0.766), whereas the additional within-study variance component was negligible. The 95% prediction interval ranged from −1.066 to 2.252, indicating substantial variability in the magnitude and direction of effects across studies and suggesting that a beneficial effect may not be observed in every clinical setting.

### 3.5. Sensitivity and Robustness Analyses

The primary finding was examined across multiple sensitivity analyses (Supplementary Table S7). Restriction to the 16 randomized controlled trials, comprising 19 effect-size estimates, yielded a statistically significant pooled effect (Hedges’ g = 0.507, 95% CI 0.141 to 0.873, *p* = 0.010), with a 95% prediction interval of −0.831 to 1.845 (Supplementary Figure S2). Exclusion of TPOMA total while retaining TPOMA balance produced a similar estimate (g = 0.596, 95% CI 0.193 to 0.999, *p* = 0.006). Restriction to BBS outcomes also yielded a statistically significant effect (g = 0.684, 95% CI 0.251 to 1.118, *p* = 0.004). Exclusion of the study with identified baseline imbalance resulted in g = 0.571 (95% CI 0.149 to 0.993, *p* = 0.011).

In contrast, exclusion of studies judged to be at high or serious risk of bias attenuated the pooled effect to *g* = 0.161 (95% CI −0.116 to 0.439, *p* = 0.217). Residual heterogeneity was also reduced in this analysis, *Q*E(11) = 17.18, *p* = 0.103, with between-study τ^2^ = 0.057.

Leave-one-study-out analyses showed that the pooled effect remained statistically significant after removal of each individual parent study (Supplementary Figure S3). The pooled estimates ranged from *g* = 0.457 to 0.645 across the 19 leave-one-study-out analyses. Xie et al. [42] exerted the largest individual influence; exclusion of this study reduced the pooled effect to *g* = 0.457 (95% CI 0.133 to 0.780, *p* = 0.009) and reduced between-study heterogeneity. Xie et al. [42] was also the only study containing an effect estimate exceeding the standardized-residual threshold of |1.96|. Exclusion of this study therefore attenuated the pooled effect but did not alter its direction or statistical significance.

### 3.6. Small-Study Effects and BBS Mean-Difference Analysis

Small-study-effect diagnostics yielded mixed findings (Supplementary Figure S4). The Egger-type regression did not indicate statistically significant funnel-plot asymmetry (standard-error coefficient = 2.237, *p* = 0.397), and the weighted regression test was likewise non-significant (*z* = 0.847, *p* = 0.397). In contrast, the Begg–Mazumdar rank-correlation test [25] was statistically significant (Kendall’s τ = 0.359, *p* = 0.019). Trim-and-fill estimated no missing studies and therefore did not alter the pooled estimate. Because these conventional diagnostics were conducted at the effect-size level and did not account fully for dependence among estimates from the same study, they were interpreted as exploratory indicators of small-study effects rather than definitive evidence of publication bias.

To express the treatment effect in clinically interpretable units, an additional analysis was restricted to the BBS. Seventeen studies contributed 18 BBS effect-size estimates. The pooled between-group mean difference in BBS change was 3.326 points in favor of vibration therapy (95% CI 1.714 to 4.938, *p* < 0.001). Residual heterogeneity remained significant, *Q*E(17) = 72.56, *p* < 0.001, with between-study τ^2^ = 5.418. The 95% prediction interval ranged from −1.952 to 8.604 BBS points, indicating considerable variation in the expected magnitude of the BBS effect across settings.

### 3.7. Moderator Analyses

Seven intervention- and patient-related moderators were evaluated using multilevel meta-regression with study-clustered CR2 inference (Table 2). Intervention duration showed a nominal association with treatment effect before correction for multiple testing, *F*(2, 10.75) = 4.33, *p* = 0.042. Relative to acute or single-session intervention (≤1 week), interventions lasting 2–4 weeks were associated with a larger effect (β = 0.738, 95% CI 0.028 to 1.449, *p* = 0.043), whereas the coefficient for interventions lasting ≥6 weeks was not statistically significant (β = 0.428, 95% CI −0.083 to 0.939, *p* = 0.088). After Benjamini–Hochberg correction, however, neither the duration omnibus test (*p*FDR = 0.293) nor the 2–4-week coefficient (*p*FDR = 0.329) remained statistically significant.

None of the other moderator omnibus tests was statistically significant. The unadjusted and FDR-adjusted *p* values were 0.623 and 0.872 for weekly treatment frequency, 0.521 and 0.872 for vibration frequency, 0.905 and 0.905 for frequency pattern, 0.159 and 0.556 for amplitude, 0.822 and 0.905 for vibration type, and 0.508 and 0.872 for stroke stage, respectively.

Residual heterogeneity remained statistically significant in all moderator models (all *p* < 0.001). Weekly treatment frequency reduced estimated between-study variance from τ^2^ = 0.586 in the primary model to 0.534, corresponding to a 9.0% reduction. Amplitude reduced between-study variance to τ^2^ = 0.503, corresponding to a 14.3% reduction, although amplitude data were available for only 14 studies contributing 17 effect-size estimates. Neither moderator was statistically significant. Intervention duration did not reduce residual between-study variance (τ^2^ = 0.623). Detailed residual heterogeneity estimates are presented in Supplementary Table S4.

Overall, none of the seven examined moderators remained statistically significant after FDR correction.

### 3.8. Dependency-Aware Subgroup Analyses

Dependency-aware subgroup estimates are presented in Table 3. Statistically significant pooled effects within individual categories were observed for chronic stroke (*g* = 0.449, 95% CI 0.103 to 0.794, *p* = 0.015), WBV (*g* = 0.612, 95% CI 0.147 to 1.077, *p* = 0.013), moderate weekly treatment frequency of 3–5 sessions per week (*g* = 0.473, 95% CI 0.045 to 0.900, *p* = 0.033), intervention duration of 2–4 weeks (*g* = 0.765, 95% CI 0.056 to 1.474, *p* = 0.037), vibration frequency ≤ 20 Hz (*g* = 0.762, 95% CI 0.025 to 1.499, *p* = 0.044), and variable-frequency protocols (*g* = 0.606, 95% CI 0.081 to 1.131, *p* = 0.029).

These within-category findings do not demonstrate differences between subgroup categories. Formal between-category inference was based on the corresponding moderator tests, and none of the seven moderators remained statistically significant after FDR correction.

For intervention duration, the acute or single-session category contained one independent study contributing two effect-size estimates and was therefore not pooled separately. Interventions lasting 2–4 weeks yielded *g* = 0.765 (95% CI 0.056 to 1.474), whereas interventions lasting ≥6 weeks yielded *g* = 0.421 (95% CI −0.073 to 0.914). The corresponding duration moderator test did not remain significant after FDR correction (*p*FDR = 0.293).

For amplitude, pooled estimates were *g* = −0.084 (95% CI −0.760 to 0.591) for <1 mm, *g* = 0.411 (95% CI −0.079 to 0.901) for 1–3.9 mm, and *g* = 0.781 (95% CI −0.650 to 2.212) for ≥4 mm. Although the point estimates increased across amplitude categories, the between-category moderator test was not statistically significant (*p* = 0.159; *p*FDR = 0.556), and the confidence intervals were wide. These findings therefore do not provide evidence of an amplitude dose–response relationship.

### 3.9. GRADE Certainty of Evidence

The certainty of evidence was assessed separately for randomized and non-randomized studies. Randomized evidence was rated as low certainty because of serious risk of bias and serious inconsistency. Eight of the 16 randomized trials were judged to be at high risk of bias, and exclusion of studies at high or serious risk of bias attenuated the pooled effect such that the confidence interval included the null. Significant residual heterogeneity and a 95% prediction interval crossing the null further indicated substantial variation in treatment effects across settings. No additional downgrade was applied for indirectness or imprecision.

Evidence from the three non-randomized studies was rated as very low certainty because of serious methodological limitations, marked inconsistency, and imprecision. The pooled estimate was Hedges’ g = 0.937 (95% CI −0.697 to 2.571; *p* = 0.261), with extreme heterogeneity (Q(2) = 57.56, *p* < 0.001; τ^2^ = 1.998) and a wide 95% prediction interval (−2.279 to 4.154). The complete GRADE evidence profile and rationale for each judgment are presented in Supplementary Table S5.

## 4. Discussion

### 4.1. Principal Findings

This domain-specific systematic review found that vibration therapy (VT) was associated with a statistically significant average improvement in validated clinical balance outcomes after stroke. In the primary dependency-aware analysis, the pooled effect was Hedges’ g = 0.593 (95% CI 0.191 to 0.995; *p* = 0.006). Restriction to randomized controlled trials (RCTs) yielded a similar statistically significant average effect (g = 0.507, 95% CI 0.141 to 0.873; *p* = 0.010), indicating that the overall result was not explained by inclusion of the three non-randomized studies. However, the 95% prediction intervals crossed the null in both the primary and RCT-only analyses, indicating substantial variation in the magnitude and direction of effects expected across clinical settings. The principal interpretation is therefore that VT is associated with a positive average effect on poststroke clinical balance, but that this benefit is not sufficiently consistent to be expected uniformly across populations, protocols, or settings.

The robustness analyses further qualify this conclusion. Leave-one-study-out analyses showed that no single study drove the overall finding: the pooled effect remained statistically significant after removal of each parent study, with estimates ranging from g = 0.457 to 0.645. Xie et al. [42] was the most influential individual study, but its exclusion still yielded a significant effect (g = 0.457, 95% CI 0.133 to 0.780; *p* = 0.009). In contrast, excluding studies judged to be at high or serious risk of bias markedly attenuated the pooled effect to g = 0.161 (95% CI −0.116 to 0.439; *p* = 0.217). This pattern suggests that the overall result is not dependent on any single study, but that its magnitude and statistical significance are sensitive to the collective methodological quality of the evidence base. Accordingly, the positive average effect should be interpreted cautiously rather than as a robust estimate that is invariant to study quality.

### 4.2. Relation to Previous Evidence

The present findings help reconcile the conflicting conclusions of previous reviews. Earlier meta-analyses reported little or no meaningful improvement in balance or mobility after WBV, particularly in chronic stroke [9,10], whereas later reviews reported statistically significant improvements in the Berg Balance Scale (BBS), fall-risk factors, or broader physical-function outcomes [11,12,13,14]. The current analysis supports the possibility of an average therapeutic benefit, but adds important qualifications by showing substantial heterogeneity, prediction intervals that cross the null, and attenuation of the effect after exclusion of studies at high or serious risk of bias. Thus, the present evidence is more consistent with a context-dependent adjunctive effect than with a large or uniformly reproducible treatment effect.

Several methodological features may explain this more qualified interpretation. First, the present review followed a construct-based protocol and restricted quantitative synthesis to validated clinical measures of dynamic or composite balance, rather than pooling clinical balance scales with instrumented postural control, functional mobility, or gait speed solely because all were described as balance-related. Second, the primary analysis used standardized between-group differences in change from baseline and a three-level random-effects model with study-clustered CR2 inference to address dependence among multiple estimates contributed by the same study. Third, extensive robustness analyses, prediction intervals, and GRADE assessments were used to evaluate the stability and certainty of the pooled estimate. These features provide a more conservative basis for interpretation than reliance on statistical significance of the average effect alone.

### 4.3. Interpretation of Intervention Parameters

None of the seven prespecified moderators remained statistically significant after false-discovery-rate (FDR) correction. Intervention duration showed a nominal association before multiplicity adjustment (*p* = 0.042), but this did not remain significant after FDR correction (pFDR = 0.293). The same was true for the 2–4-week coefficient, which lost statistical significance after adjustment. Therefore, the current data do not establish intervention duration as an independent treatment-effect modifier and do not support identifying 2–4 weeks as an optimal treatment period. The favorable pooled effect observed within the 2–4-week subgroup should instead be regarded as a hypothesis-generating signal that requires confirmation in prospectively designed dose-ranging trials.

The subgroup analyses require similar caution. Significant pooled effects within categories such as chronic stroke, WBV, 3–5 sessions per week, 2–4 weeks of treatment, vibration frequency ≤20 Hz, or variable-frequency protocols indicate that the pooled effect within those categories differed from zero; they do not demonstrate that those categories were more effective than their alternatives. Formal between-category inference was based on the corresponding moderator tests, and none remained statistically significant after FDR correction. Consequently, the current evidence does not establish that stroke stage, vibration type, weekly treatment frequency, nominal vibration frequency, frequency pattern, or treatment duration identifies a superior protocol.

Amplitude also should not be interpreted as demonstrating a dose–response relationship. Although point estimates increased across amplitude categories, the formal moderator test was not statistically significant (*p* = 0.159; pFDR = 0.556), and confidence intervals were wide. In addition, reported displacement is not mechanically equivalent across devices: studies may report peak displacement, peak-to-peak displacement, or device-specific settings, while the acceleration transmitted to the body depends jointly on displacement, frequency, posture, loading, and platform configuration. The observed amplitude pattern is therefore descriptive rather than evidence of a confirmed dose–response relationship. More generally, the lack of statistically significant moderation should not be interpreted as proof of equivalence because several categories contained relatively few independent studies and moderator analyses remained underpowered.

### 4.4. Potential Mechanisms

Several complementary mechanisms could plausibly contribute to poststroke balance improvement with vibration. Mechanical vibration activates muscle-spindle Ia afferents, facilitates tonic vibration reflex responses, and may increase recruitment of muscles involved in ankle, knee, and hip stabilization [5]. Localized vibration may modulate spinal excitability through presynaptic and reciprocal inhibitory pathways, potentially reducing abnormal reflex activity and improving joint control [45]. Plantar vibration may augment cutaneous and proprioceptive information from the support surface and thereby improve postural orientation and detection of weight shifts [46].

Repeated vibration may also influence supraspinal sensorimotor processing. Experimental studies have reported modulation of cortical activity during gait or ankle movement after WBV, and repeated focal muscle vibration has been associated with longer-term changes in cortical excitability [7,33,47]. These mechanisms could enhance integration of somatosensory input with postural motor commands. However, the present meta-analysis did not demonstrate that any specific vibration dose, duration, frequency, or amplitude produces these mechanisms to a greater extent, nor did it test whether neurophysiological changes mediate clinical balance improvement. These mechanisms should therefore be viewed as biologically plausible hypotheses that can inform future mechanistic and dose-ranging studies rather than as explanations for confirmed moderator effects.

### 4.5. Clinical Implications

VT should currently be considered a potential adjunct to, rather than a substitute for, active and task-specific balance rehabilitation. The additional BBS analysis provides a clinically interpretable estimate: across 17 studies contributing 18 effect-size estimates, the mean improvement in BBS change was 3.326 points greater with VT than with control (95% CI 1.714 to 4.938; *p* < 0.001). However, the 95% prediction interval ranged from −1.952 to 8.604 points, indicating that the expected BBS effect may vary substantially across clinical settings. Moreover, this pooled mean difference should not be interpreted as demonstrating that the average participant exceeded a particular minimal clinically important difference, because clinically important thresholds vary according to population, baseline severity, recovery stage, and method of estimation.

The evidence does not currently support a universal setting for vibration duration, frequency, amplitude, frequency pattern, or modality. This is reinforced by the attenuation of the pooled effect after exclusion of studies at high or serious risk of bias and by the absence of statistically significant moderators after multiplicity correction. Clinicians considering VT should therefore prioritize safety, patient tolerance, the feasibility of the associated posture or exercise, and reproducible documentation of the delivered mechanical dose, while evaluating individual response using validated clinical outcomes. The current evidence is insufficient to recommend a specific VT protocol for routine poststroke balance rehabilitation.

### 4.6. Strengths and Limitations

This review has several strengths. It was prospectively registered and grounded in a peer-reviewed protocol, and deviations from that protocol were reported explicitly. The construct-based framework reduced conceptual heterogeneity by restricting the primary synthesis to validated clinical balance scales. The final analysis standardized effect-size derivation to between-group change from baseline and used a three-level random-effects model with study-clustered CR2 robust inference to address dependent estimates. Multiple sensitivity and robustness analyses evaluated study design, risk of bias, instrument choice, outcome definition, baseline imbalance, pre-post correlation assumptions, within-study dependence, outliers, and individual study influence. The use of FDR correction reduced the risk of overinterpreting multiple moderator tests, while the additional BBS mean-difference analysis improved clinical interpretability. Finally, RoB 2, ROBINS-I, and GRADE were integrated so that effect magnitude, methodological quality, heterogeneity, and certainty could be interpreted together.

Several limitations remain. First, substantial between-study heterogeneity persisted in the primary and RCT-only analyses, and both prediction intervals crossed the null. This indicates that a positive average effect does not guarantee benefit in a new clinical setting. Second, methodological quality materially influenced the pooled result: exclusion of studies at high or serious risk of bias reduced the effect and rendered it statistically non-significant. Half of the RCTs were judged to be at high risk of bias, most commonly because randomization or allocation concealment was insufficiently described, while the non-randomized studies were additionally vulnerable to confounding. Third, the evidence base was modest relative to the number of candidate moderators. Even with FDR correction, study-level meta-regression is vulnerable to ecological confounding and limited statistical power; therefore, the absence of significant moderation does not establish equivalence among treatment parameters.

Fourth, vibration protocols and reporting conventions varied markedly, and amplitude, displacement convention, calculated acceleration, actual exposure time, posture, and adherence were not reported consistently. Fifth, multiple effect sizes from the same studies and shared comparator groups created statistical dependence. Although the multilevel model and study-clustered CR2 inference were used to address this issue, a complete sampling variance-covariance matrix could not be reconstructed for all studies. Sixth, change-score variances required assumed pre-post correlations when not directly reported, although alternative assumptions were examined in sensitivity analyses. Seventh, the completed database set differed from the published protocol because Scopus was not searched and MEDLINE was searched separately, which may have reduced retrieval of otherwise eligible reports despite citation searching. Eighth, the primary synthesis focused on clinical dynamic/composite balance and therefore does not directly generalize to instrumented postural control, functional mobility, gait performance, falls, or long-term participation. Finally, small-study-effect diagnostics were mixed: regression-based tests were non-significant, the Begg–Mazumdar test was significant, and trim-and-fill estimated no missing studies. Because these conventional diagnostics do not fully account for dependent effect sizes, publication bias cannot be confirmed or excluded. In addition, complete bibliographic traceability of the 15 reports recorded as not retrieved during the original screening process could not be reconstructed retrospectively, limiting verification of report-specific retrieval attempts.

### 4.7. Implications for Research

Future trials should prioritize methodological rigor before refinement of a preferred vibration protocol. Adequately powered, prospectively registered RCTs with concealed allocation, assessor blinding, credible sham vibration or an equivalent co-intervention control, and prespecified outcome analysis are needed. Reporting should include device type, oscillation direction, frequency, displacement definition, calculated acceleration, body position, knee angle, footwear, exercise task, session duration, rest intervals, sessions per week, total sessions, treatment duration, adherence, adverse events, stroke stage, baseline balance severity, and concurrent rehabilitation dose. Such standardization would permit more valid comparison of mechanical exposure across devices and studies.

Dose-ranging, factorial, or adaptive trials are particularly important because the present moderator analyses did not identify any treatment parameter that remained statistically significant after multiplicity correction. These studies should manipulate duration, weekly frequency, vibration frequency, amplitude, or total mechanical exposure while holding relevant co-interventions constant. Future research should also report outcomes in both standardized form and clinically interpretable scale units, include follow-up assessments to determine durability, and distinguish clinical balance from instrumented postural control, gait, falls, and patient-reported outcomes. Individual-participant-data meta-analysis may subsequently help determine whether baseline balance severity, sensory impairment, spasticity, stroke stage, or capacity for active exercise modifies treatment response.

## 5. Conclusions

Vibration therapy was associated with a statistically significant average improvement in validated clinical balance outcomes after stroke (Hedges’ g = 0.593), and the effect remained significant when the analysis was restricted to randomized controlled trials (g = 0.507). However, substantial between-study heterogeneity and prediction intervals crossing the null indicate that the magnitude and direction of benefit are likely to vary across clinical settings. Moreover, exclusion of studies at high or serious risk of bias markedly attenuated the pooled effect and rendered it statistically non-significant, indicating that the estimated benefit is sensitive to study quality. None of the seven prespecified moderators remained statistically significant after false-discovery-rate correction, and the available evidence therefore does not support an optimal treatment duration, frequency, amplitude, vibration type, frequency pattern, or stroke stage. The BBS-specific analysis showed a mean between-group improvement of 3.326 points, but its wide prediction interval also indicated substantial variation across settings. Overall, VT may provide an adjunctive benefit for poststroke balance rehabilitation, but the low certainty of randomized evidence and very low certainty of non-randomized evidence preclude recommendation of a specific protocol. Well-designed dose-ranging randomized trials with standardized mechanical-dose reporting and clinically meaningful follow-up outcomes are needed.

## Figures and Tables

**Table 1:** Study characteristics of 19 studies selected for the meta-analysis.

Author & Year	Type of Study	Participants	Sample (N, Age)	Intervention & Control	Outcome Measures
Annino et al., 2025 [28]	RCT	Chronic	I:19 (67.8 ± 8.3), C:18 (69.4 ± 10.4)	LMV + conventional physical therapy vs. conventional physical therapy	BBS
Brogårdh et al., 2012 [16]	RCT	Chronic	I:16 (61.3 ± 8.5), C:15 (63.9 ± 5.8)	WBV vs. sham WBV	BBS, TUG
Kim et al., 2021 [29]	RCT	Postacute	I:20 (57.2 ± 11.0), C:18 (55.7 ± 10.4)	WBV + squat training vs. squat training	BBS, TUG, Functional Ambulation Category (FAC)
Lau et al., 2012 [30]	RCT	Chronic	I:41 (57.3 ± 11.3), C:41 (57.4 ± 11.1)	WBV + exercise vs. exercise	BBS, Activities-specific Balance Confidence Scale, Movement velocity (MVL)
Lee et al., 2015 [31]	RCT	Chronic	I:12 (59.3 ± 13.2), C:9 (56.0 ± 9.1)	Horizontal WBV + rehabilitation vs. rehabilitation	BBS, TUG
Lee et al., 2017 [32]	RCT	Subacute	I:15 (59.1 ± 16.9), C:15 (64.4 ± 14.8)	WBV + physical therapy vs. physical therapy	BBS, Functional Ambulation Category (FAC), Trunk Impairment Scale (TIS)
Lee et al., 2020 [33]	RCT	Chronic	I:15 (58.7 ± 9.1), C:14 (53.4 ± 9.6)	WBV vs. sham WBV	TPOMA balance, gait, and total; TUG
Liao et al., 2016 [34]	RCT	Chronic	I1:28 (60.8 ± 8.3), I2:28 (62.9 ± 10.2), C:28 (59.8 ± 9.1)	Low-intensity WBV or High-intensity WBV + exercise vs. exercise	Mini-BESTest, TUG, Activities-specific Balance Confidence Scale (ABC)
Majeed et al., 2024 [35]	RCT	Chronic	I:16 (63.9 ± 6.9), C:16 (63.4 ± 7.9)	Plantar vibration + physical therapy vs. physical therapy	BBS, Modified Postural Assessment Scale for Stroke (MPASS)
Marín et al., 2013 [36]	RCT	Chronic	I:11 (62.3 ± 10.6), C:9 (64.4 ± 7.6)	WBV + isometric rehabilitation vs. isometric rehabilitation	BBS
Merkert et al., 2011 [37]	RCT	Postacute	I:25 (74.5 ± 8.3), C:23 (74.5 ± 8.6)	WBV + geriatric rehabilitation vs. geriatric rehabilitation	BBS, TUG, Tinetti Gait Test
Nagino et al., 2013 [38]	RCT	Chronic	I:15 (68.9 ± 11.6), C:15 (70.2 ± 7.7)	WBV + rehabilitation vs. rehabilitation	BBS, Functional Reach Test (FRT)
Sade et al., 2020 [39]	RCT	Chronic	I:26 (46.8 ± 15.0), C:17 (51.6 ± 10.0)	WBV + rehabilitation vs. rehabilitation	BBS, TUG
van Nes et al., 2006 [15]	RCT	Postacute	I:27 (59.7 ± 12.3), C:26 (62.6 ± 7.6)	WBV + exercise vs. exercise	BBS, Trunk Control Test
Wei et al., 2022 [40]	RCT	Chronic	I1:26 (72.4 ± 5.9), I2:26 (70.2 ± 5.1), C:26 (71.9 ± 6.0)	WBV + physiotherapy vs. physiotherapy	BBS
Xiao et al., 2022 [41]	RCT	Chronic	I:25 (63.5 ± 5.3), C:25 (63.6 ± 4.2)	WBV + ESWT + conventional treatment vs. sham WBV + ESWT + conventional treatment	BBS
Xie et al., 2021 [42]	Retrospective controlled study	Subacute	I:65 (60.4 ± 6.4), C:65 (59.8 ± 6.6)	WBV + routine rehabilitation vs. routine rehabilitation	BBS, TUG, Maximum Walking Speed, Functional Reach Test
Xu et al., 2024 [43]	Prospective cohort study	Chronic	I:17 (58.9 ± 9.7), C:18 (54.2 ± 10.8)	WBV + rehabilitation vs. rehabilitation	BBS, Functional Reach Test (FRT), Romberg Test Length (RTL), Romberg Test Area (RTA)
Yoon et al., 2024 [44]	Retrospective controlled study	Subacute	I:22 (64.7 ± 8.8), C:22 (67.0 ± 8.9)	Proprioceptive body vibration + conventional physical therapy vs. conventional physical therapy	BBS, Trunk Impairment Scale (TIS)

Abbreviations: WBV, whole-body vibration; LMV, local muscle vibration; ESWT, extracorporeal shock wave therapy; BBS, Berg Balance Scale; TPOMA, Tinetti Performance-Oriented Mobility Assessment. Chronic stroke was defined as stroke onset >6 months, whereas subacute/postacute stroke included participants within 6 months after stroke onset. For multi-arm trials (e.g., Liao et al. [34] and Wei et al. [40]), different vibration frequencies were treated as separate intervention arms. Table 1 lists all reported balance-related outcomes; the primary quantitative synthesis used BBS, Mini-BESTest, and TPOMA balance or total outcomes. Detailed vibration parameters, treatment schedules, posture or stimulation site, and device information are provided in Supplementary Table S3.

**Table 2:** Dependency-aware multilevel meta-regression analyses of potential moderators of clinical balance outcomes.

Moderator	Studies	Effect Sizes	F (df1, df2)	Raw *p*	FDR *p*
Duration	19	22	4.33 (2, 10.75)	0.042	0.293
Frequency per week	19	22	0.55 (2, 3.15)	0.623	0.872
Frequency (Hz)	19	22	0.52 (1, 3.24)	0.521	0.872
Frequency pattern	19	22	0.01 (1, 14.95)	0.905	0.905
Amplitude	14	17	2.65 (2, 5.33)	0.159	0.556
Vibration type	19	22	0.06 (1, 2.79)	0.822	0.905
Disease stage	19	22	0.47 (1, 9.84)	0.508	0.872

Note. Studies denotes the number of independent parent studies; effect sizes denotes the number of contributing effect-size estimates. Moderator tests were obtained from multilevel REML meta-regression models with study-clustered CR2 inference. Benjamini–Hochberg false discovery rate (FDR) correction was applied across the seven omnibus moderator tests. Amplitude data were available for 14 studies contributing 17 effect-size estimates. Category-specific coefficients and residual heterogeneity statistics are provided in Supplementary Table S4.

**Table 3:** Dependency-aware subgroup analyses of vibration therapy effects on clinical balance outcomes after stroke.

Moderator	Category	Studies	ES	Hedges’ g (95% CI)	*p*	Moderator Raw *p*	FDR *p*
Disease stage	Chronic	13	16	0.449 (0.103, 0.794)	0.015	0.508	0.872
Disease stage	Subacute/Postacute	6	6	0.845 (−0.459, 2.149)	0.157	0.508	0.872
Vibration type	WBV	16	19	0.612 (0.147, 1.077)	0.013	0.822	0.905
Vibration type	LV	3	3	0.488 (−1.241, 2.216)	0.348	0.822	0.905
Frequency/week	≤2 sessions/week	3	4	0.319 (−1.165, 1.802)	0.445	0.623	0.872
Frequency/week	3–5 sessions/week	13	15	0.473 (0.045, 0.900)	0.033	0.623	0.872
Frequency/week	≥6 sessions/week	3	3	1.278 (−1.842, 4.398)	0.22	0.623	0.872
Duration	≤1 week	1	2	Not pooled	-	0.042	0.293
Duration	2–4 weeks	10	12	0.765 (0.056, 1.474)	0.037	0.042	0.293
Duration	≥6 weeks	8	8	0.421 (−0.073, 0.914)	0.083	0.042	0.293
Frequency	≤20 Hz	10	11	0.762 (0.025, 1.499)	0.044	0.521	0.872
Frequency	>20 Hz	11	11	0.311 (−0.024, 0.646)	0.065	0.521	0.872
Amplitude	<1 mm	3	4	−0.084 (−0.760, 0.591)	0.589	0.159	0.556
Amplitude	1–3.9 mm	6	7	0.411 (−0.079, 0.901)	0.083	0.159	0.556
Amplitude	≥4 mm	5	6	0.781 (−0.650, 2.212)	0.204	0.159	0.556
Frequency pattern	Fixed	11	14	0.579 (−0.074, 1.233)	0.077	0.905	0.905
Frequency pattern	Variable	8	8	0.606 (0.081, 1.131)	0.029	0.905	0.905

Note. Studies denotes the number of independent parent studies; ES denotes the number of effect-size estimates. Subgroup-specific *p* values test whether the pooled effect within each category differs from zero and should not be interpreted as tests of differences between categories. Between-category differences were evaluated using the corresponding multilevel meta-regression models with study-clustered CR2 inference. FDR-adjusted *p* values were obtained using the Benjamini–Hochberg procedure across the seven moderator tests. WBV, whole-body vibration; LV, local vibration; CI, confidence interval.

## Data Availability

The extracted effect-size-level dataset used in the primary meta-analysis is provided in Supplementary Table S6, and the sensitivity and robustness results are summarized in Supplementary Table S7. Additional analytic files, including the R code used for the primary and sensitivity analyses and documentation of effect-size calculations and covariance assumptions, are available from the corresponding author upon reasonable request.

## Supplementary Materials

The following supporting information can be downloaded at: https://www.mdpi.com/article/10.3390/healthcare14193307/s1, Figure S1. Risk of bias assessment for the included studies; Figure S2. Forest plot of the sensitivity analysis restricted to randomized controlled trials (RCTs); Table S1. Complete database-specific search strategies used in the systematic review; Table S2. Adverse events, withdrawals, and treatment adherence reported in the included studies; Table S3. Detailed vibration-intervention characteristics of the included studies; Table S4. Residual heterogeneity following multilevel moderator analyses; Figure S3. Leave-one-study-out analysis of the primary pooled effect; Table S5. GRADE evidence profile of vibration therapy compared with control interventions for clinical balance outcomes after stroke; Table S6. Effect-size-level data contributing to the primary meta-analysis; Table S7. Sensitivity and robustness analyses of the pooled effect of vibration therapy on clinical balance outcomes; Figure S4. Funnel plot of the effect-size estimates contributing to the primary meta-analysis; Figure S5. Forest plot of the BBS raw mean-difference analysis.

## Abbreviations

ABC	Activities-specific Balance Confidence Scale
BBS	Berg Balance Scale
CI	Confidence interval
FMV	Focal muscle vibration
GRADE	Grading of Recommendations Assessment, Development and Evaluation
LMV/LV	Local muscle vibration/local vibration
Mini-BESTest	Mini-Balance Evaluation Systems Test
PRISMA	Preferred Reporting Items for Systematic Reviews and Meta-Analyses
ROBINS-I	Risk of Bias in Non-randomized Studies of Interventions
RoB 2	Cochrane Risk of Bias 2
TPOMA	Tinetti Performance-Oriented Mobility Assessment
TUG	Timed Up and Go
VT	Vibration therapy
WBV	Whole-body vibration
