# Supplementary material for: Vibration Therapy for Poststroke Clinical Balance: A Domain-Specific Systematic Review and Multilevel Meta-Analysis
Source: Healthcare (Basel). 2026 Oct 3;14(19):3307. doi: 10.3390/healthcare14193307 (PMC13665116; doi:10.3390/healthcare14193307)
Supplement: Supplementary file 1 [file healthcare-14-03307-s001.zip › healthcare-4543759-supplementary.pdf]

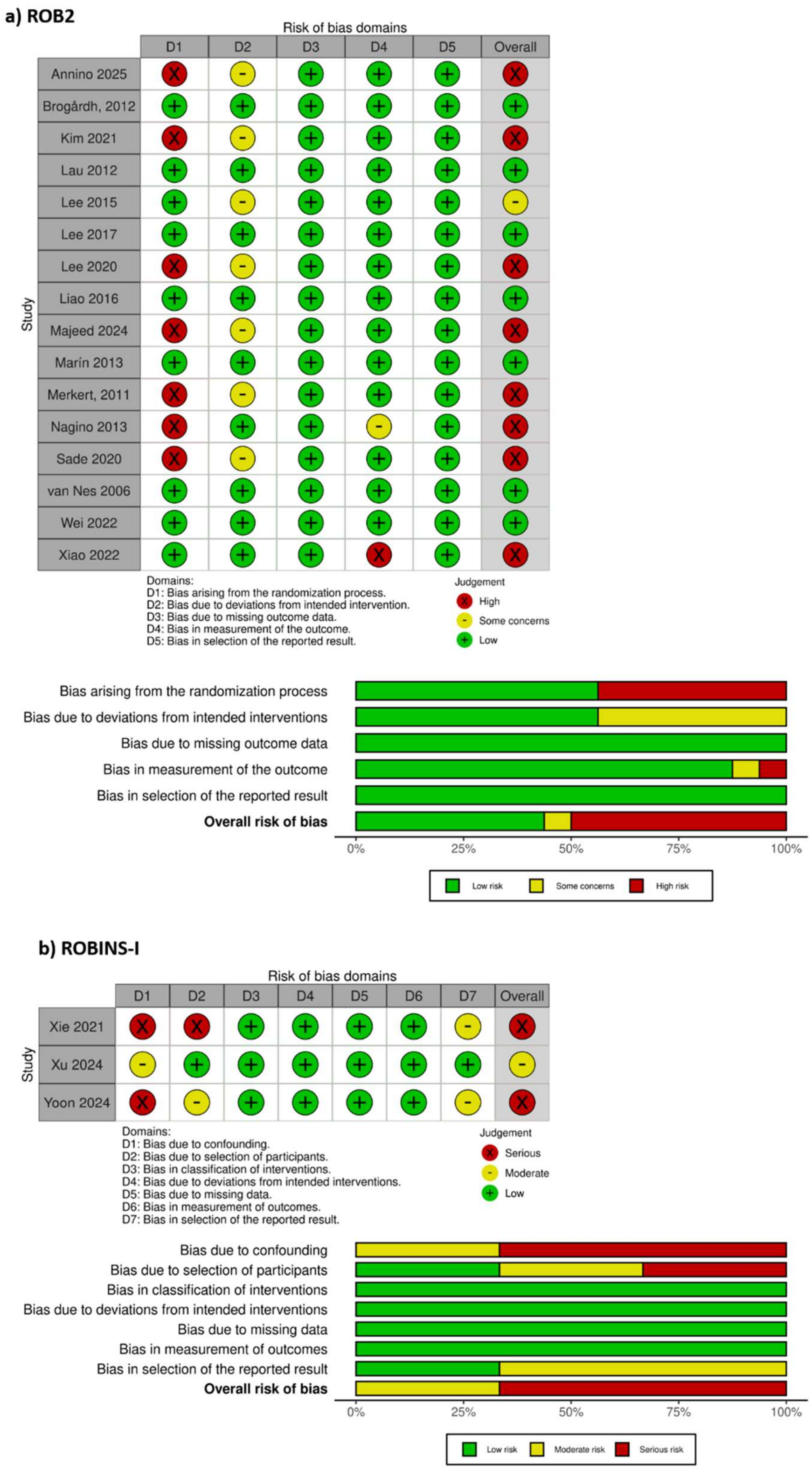

Figure S1. Risk of bias assessment for the included studies.

a) Risk of bias summary and traffic-light plot for randomized controlled trials using the RoB 2 tool; b) Risk of bias summary and traffic-light plot for non-randomized studies using the ROBINS-I tool.

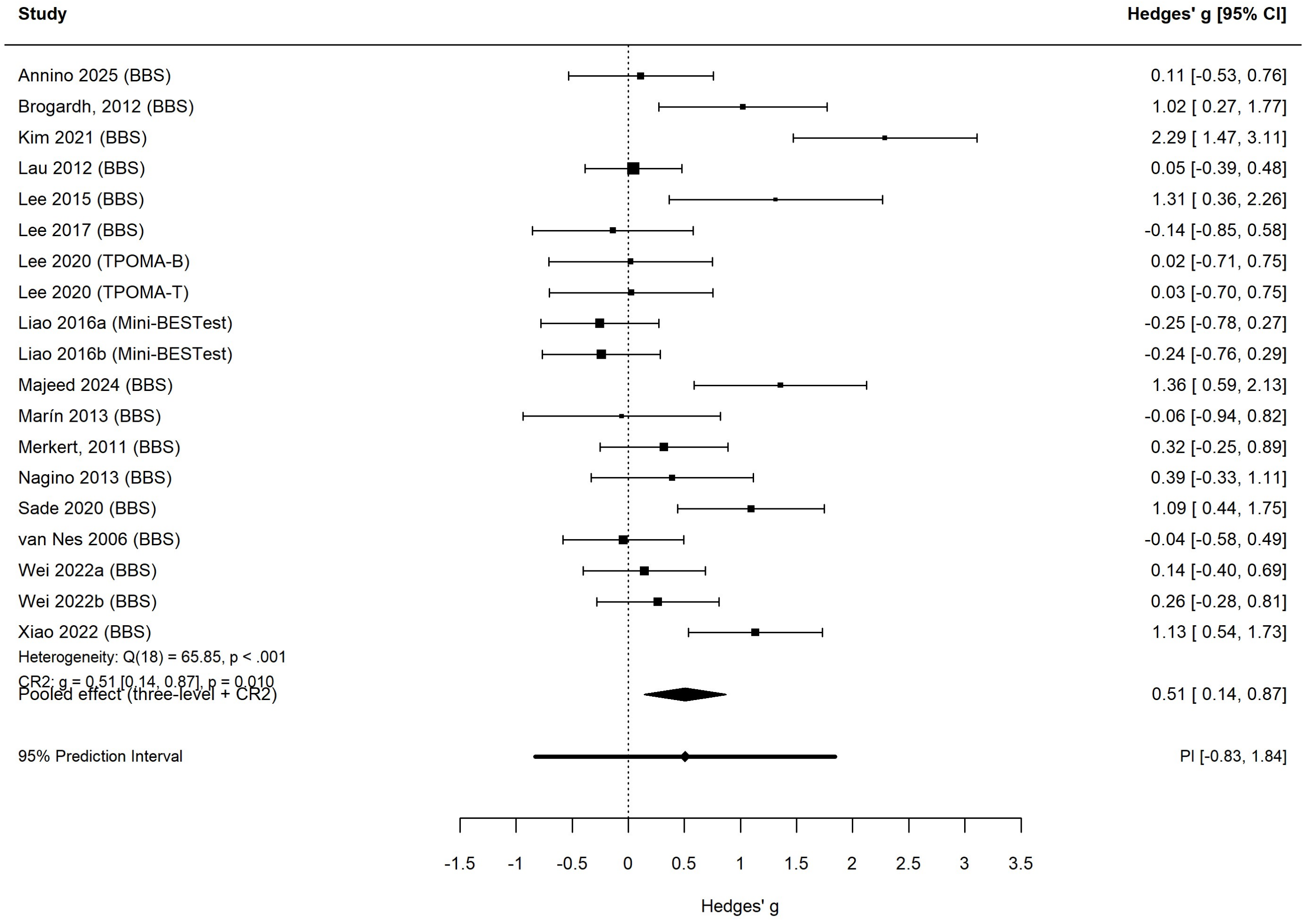

Figure S2. Forest plot of the sensitivity analysis restricted to randomized controlled trials (RCTs).

The analysis included 19 effect-size estimates from 16 randomized controlled trials after exclusion of the three non-randomized studies. Squares and horizontal lines represent individual Hedges' *g* estimates and their 95% confidence intervals (CIs), respectively. The pooled estimate was obtained using a three-level random-effects model with study-clustered CR2 inference (Hedges' $g = 0.507$, 95% CI 0.141 to 0.873, $p = 0.010$). The 95% prediction interval ranged from -0.831 to 1.845.

Table S1. Complete database-specific search strategies used in the systematic review.

Database	Platform / interface	Date searched	Fields	Complete search strategy	Filters / limits	Records retrieved
PubMed	PubMed, National Library of Medicine	[31 May 2026]	MeSH; Title/Abstract	#1 Stroke: "Stroke"[Mesh] OR stroke*[Title/Abstract] OR "post-stroke"[Title/Abstract] OR "post stroke"[Title/Abstract] OR cerebrovascular[Title/Abstract] OR hemipleg*[Title/Abstract] OR hemipar*[Title/Abstract] OR paresis[Title/Abstract] #2 Vibration: "Vibration"[Mesh] OR vibration*[Title/Abstract] OR "vibration therapy"[Title/Abstract] OR "vibration training"[Title/Abstract] OR "vibration intervention"[Title/Abstract] OR "whole body vibration"[Title/Abstract] OR "whole-body vibration"[Title/Abstract] OR WBV[Title/Abstract] OR "focal vibration"[Title/Abstract] OR "focal muscle vibration"[Title/Abstract] OR FMV[Title/Abstract] OR "local vibration"[Title/Abstract] OR "segmental vibration"[Title/Abstract] #3 Balance/postural control: "Postural Balance"[Mesh] OR balance[Title/Abstract] OR "postural balance"[Title/Abstract] OR "postural control"[Title/Abstract] OR "postural stability"[Title/Abstract] OR equilibrium[Title/Abstract] OR stability[Title/Abstract] OR "balance function"[Title/Abstract] OR "balance ability"[Title/Abstract] OR gait[Title/Abstract] #4: #1 AND #2 AND #3	From inception to 31 March 2026; no study-design or language filter	118
MEDLINE	Ovid MEDLINE	[31 May 2026]	MeSH; Title/Abstract (.ti,ab.)	1. exp Stroke/ 2. (stroke* or post-stroke or poststroke or "post stroke" or cerebrovascular or hemipleg* or hemipar* or paresis).ti,ab. 3. 1 or 2 4. exp Vibration/ 5. (vibration* or "vibration therapy" or "vibration training" or "vibration intervention" or "whole body vibration" or "whole-body vibration" or WBV or "focal vibration" or "focal muscle vibration" or FMV or "local vibration" or "segmental vibration").ti,ab. 6. 4 or 5 7. exp Postural Balance/ 8. (balance or "postural balance" or "postural control" or "postural stability" or equilibrium or stability or "balance function" or "balance ability" or gait).ti,ab. 9. 7 or 8 10. 3 and 6 and 9	From inception to 31 March 2026; no study-design or language filter	67
Embase	Embase.com	[31 May 2026]	Emtree; Title/Abstract	#1 Stroke: 'cerebrovascular accident'/exp OR stroke*:ti,ab OR 'post-stroke':ti,ab OR poststroke:ti,ab OR 'post stroke':ti,ab OR cerebrovascular:ti,ab OR hemipleg*:ti,ab OR hemipar*:ti,ab OR paresis:ti,ab #2 Vibration: 'vibration'/exp OR vibration*:ti,ab OR 'vibration therapy':ti,ab OR 'vibration training':ti,ab OR 'vibration intervention':ti,ab OR 'whole body vibration':ti,ab OR 'whole-body vibration':ti,ab OR WBV:ti,ab OR 'focal vibration':ti,ab OR 'focal muscle vibration':ti,ab OR FMV:ti,ab OR 'local vibration':ti,ab OR 'segmental vibration':ti,ab #3 Balance/postural control: 'body equilibrium'/exp OR balance:ti,ab OR	From inception to 31 March 2026; no study-design or language filter	156

				'postural balance':ti,ab OR 'postural control':ti,ab OR 'postural stability':ti,ab OR equilibrium:ti,ab OR stability:ti,ab OR 'balance function':ti,ab OR 'balance ability':ti,ab OR gait:ti,ab #4: #1 AND #2 AND #3		
Cochrane Library	Cochrane Library, Wiley	[5 June 2026]	MeSH; Title/Abstract/Keyword (ti,ab,kw)	#1 MeSH descriptor: [Stroke] explode all trees #2 (stroke* OR "post-stroke" OR poststroke OR "post stroke" OR cerebrovascular OR hemipleg* OR hemipar* OR paresis):ti,ab,kw #3 #1 OR #2 #4 MeSH descriptor: [Vibration] explode all trees #5 (vibration* OR "vibration therapy" OR "vibration training" OR "vibration intervention" OR "whole body vibration" OR "whole-body vibration" OR WBV OR "focal vibration" OR "focal muscle vibration" OR FMV OR "local vibration" OR "segmental vibration"):ti,ab,kw #6 #4 OR #5 #7 MeSH descriptor: [Postural Balance] explode all trees #8 (balance OR "postural balance" OR "postural control" OR "postural stability" OR equilibrium OR stability OR "balance function" OR "balance ability" OR gait):ti,ab,kw #9 #7 OR #8 #10 #3 AND #6 AND #9	From inception to 31 March 2026; no study-design or language filter	122
Web of Science Core Collection	Web of Science, Clarivate	[5 June 2026]	Topic (TS)	#1 TS=(stroke* OR "post-stroke" OR poststroke OR "post stroke" OR cerebrovascular OR hemipleg* OR hemipar* OR paresis) #2 TS=(vibration* OR "vibration therapy" OR "vibration training" OR "vibration intervention" OR "whole body vibration" OR "whole-body vibration" OR WBV OR "focal vibration" OR "focal muscle vibration" OR FMV OR "local vibration" OR "segmental vibration") #3 TS=(balance OR "postural balance" OR "postural control" OR "postural stability" OR equilibrium OR stability OR "balance function" OR "balance ability" OR gait) #4 #1 AND #2 AND #3	From inception to 31 March 2026; no study-design or language filter	98
Total	—	—	—	—	—	561

Abbreviations: FMV, focal muscle vibration; MeSH, Medical Subject Headings; TS, Topic; WBV, whole-body vibration.

Note: Searches covered each database from inception through 31 March 2026. The 'Date searched' column records the date on which each search was executed; the search coverage/cutoff remained 31 March 2026. No randomized controlled trial/study-design filter or language restriction was applied at the database-search stage. Controlled vocabulary and database-specific syntax were adapted to the respective search platforms. Scopus, which was specified in the published protocol, was not included in the final search, whereas MEDLINE was searched separately. The contemporaneous search documentation does not record a more specific rationale for this implementation deviation; it is therefore reported transparently rather than retrospectively inferred. This deviation is described in Sections 2.1.1 and 2.3 of the main manuscript.

Table S2. Adverse events, withdrawals, and treatment adherence reported in the included studies.

Study	Adverse events / safety	Withdrawals or loss to follow-up	Intervention-related withdrawal	Adherence / compliance
van Nes et al., 2006 [1]	No adverse reactions occurred during or directly after treatment. Treatment was generally well tolerated.	WBV was stopped prematurely in one participant because of severe shoulder pain, although the authors stated that this could not be directly related to WBV. Two participants in the control group were lost to follow-up (second cerebral infarction, n=1; refusal to continue, n=1).	Uncertain. One WBV participant stopped because of severe shoulder pain, but a direct relationship with WBV was not established.	Intervention exposure was reported (mean 26.2 ± 3.9 sessions/hours as reported in the study); formal adherence percentage NR.
Merkert et al., 2011 [2]	NR. No explicit study-level adverse-event statement was identified.	66 enrolled; 48 completed (25 intervention, 23 control). Discontinuations were attributed to early discharge, hospital transfer, or deterioration in health.	Not reported as intervention-related.	Completion reported; formal adherence/compliance rate NR.
Brogårdh et al., 2012 [3]	No discernible discomfort was reported; 15 participants experienced transient mild muscle soreness or muscle fatigue, irrespective of platform assignment.	All 31 randomized participants completed the 6-week training.	None reported.	All participants completed the prescribed 12-session, 6-week WBV program.
Lau et al., 2012 [4]	No severe adverse effects. Three participants reported mild dizziness during WBV; four reported lower-limb soreness/fatigue, including two in the WBV group. Symptoms gradually subsided after the first few sessions.	Six participants (3/group) dropped out for reasons explicitly reported as unrelated to the training; 76 completed training and follow-up.	None reported.	76 completed the program. Time required to complete 24 sessions and maximum interval between sessions were reported and did not differ between groups.
Marín et al., 2013 [5]	No side effects or vibration-related adverse effects were reported.	No loss to follow-up or intervention discontinuation in either group.	None.	All randomized participants completed the study (11 experimental; 9 sham).
Nagino et al., 2013 [6]	No adverse reactions occurred during or directly after WBV; no serious adverse events were reported.	All participants underwent baseline and post-intervention assessment.	None reported.	All WBV participants completed the entire program.

Lee., 2015 [7]	No pain, dizziness, nausea, or other discomfort was documented during WBV. However, the authors explicitly acknowledged that adverse effects were not investigated clearly.	One WBV participant was discharged. In the control group, one participant dropped out after a hip fracture due to a fall and three were discharged.	None explicitly attributed to WBV.	Formal adherence/compliance NR.
Liao et al., 2016 [8]	One low-intensity WBV participant reported mild knee pain; five participants reported fatigue (3 low-intensity, 2 high-intensity).	84 randomized; 74 completed. Ten participants withdrew/dropped out (4 high-intensity WBV, 5 low-intensity WBV, 1 control).	Yes. The participant with knee pain and five participants reporting fatigue subsequently dropped out.	Missed sessions were rescheduled to facilitate completion of all 30 sessions. Among completers, time required to complete the 30 sessions and intervals between sessions were similar across groups.
Lee et al., 2017 [9]	NR.	All 30 participants (15/group) completed the 2-week therapy.	None reported.	Complete treatment participation reported; formal adherence percentage NR.
Lee et al., 2020 [10]	One participant reported fatigue. No other serious adverse effect was reported during or after the experimental session.	One control-group participant dropped out because of fatigue; 29 participants completed the study (15 WBV, 14 control).	No WBV-related withdrawal reported; the fatigue-related withdrawal occurred in the control group.	29/30 completed the study; formal adherence percentage NR.
Sade et al., 2020 [11]	No adverse effects from WBV; treatment was reported as well tolerated.	Three control participants discontinued for social reasons; 43 completed (26 WBV, 17 control). No WBV participant withdrew.	None.	Authors explicitly reported that compliance was “excellent”; all WBV participants completed the intervention.
Kim et al., 2021 [12]	NR.	Two control-group participants were lost to follow-up because of health problems; 38 completed (20 WBV, 18 control).	None reported for WBV.	All 20 WBV participants completed therapy; formal adherence percentage NR.
Xie et al., 2021 [13]	Specific adverse-event data NR. The authors described WVT as safe in their discussion, but no systematic adverse-event results were provided.	Not applicable/not specifically reported as prospective treatment withdrawal because this was a retrospective analysis of 130 treated patients.	NR.	NR.
Wei et al., 2022 [14]	No adverse events were reported during the study.	No intervention-related withdrawal was reported in the available study report.	None reported.	Ten-session intervention was described; formal adherence/compliance percentage NR.

Xiao et al., 2022 [15]	NR. No explicit study-level adverse-event statement was identified.	Withdrawal/dropout information was not clearly reported.	NR.	NR.
Majeed et al., 2024 [16]	NR.	36 randomized (18/group); four participants dropped out and 32 completed the study. Reasons for dropout were not reported in the text available.	NR.	32/36 completed; formal adherence/compliance rate NR.
Xu et al., 2024 [17]	NR.	No clearly reported intervention-related withdrawal information was identified.	NR.	NR.
Yoon et al., 2024 [18]	NR. No explicit adverse-event results for the study intervention were identified.	No clearly reported intervention-related withdrawals were identified in the study report.	NR.	Formal adherence/compliance data NR.
Annino et al., 2025 [19]	No adverse effects following LMV treatment were reported.	Three of 37 participants dropped out because of personal circumstances (2 CPT+LMV; 1 CPT); 34 completed all training sessions.	None reported.	34 participants completed all training sessions; formal adherence percentage NR.

Abbreviations: CPT, conventional physiotherapy; HWBV, high-intensity whole-body vibration; LMV, localized muscle vibration; LWBV, low-intensity whole-body vibration; NR, not reported; WBV, whole-body vibration; WVT, whole-body vibration training.

Note: "NR" indicates that the corresponding information was not explicitly reported in the published article. Absence of reported adverse events was not interpreted as evidence that no adverse events occurred. Withdrawals were classified as intervention-related only when the original report explicitly attributed discontinuation to an adverse symptom or the intervention.

1. van Nes IJW, Latour H, Schils F, et al. Long-term effects of 6-week whole-body vibration on balance recovery and activities of daily living in the postacute phase of stroke: a randomized, controlled trial. Stroke. 2006;37(9):2331-2335. doi:10.1161/01.STR.0000236494.62957.f3.
2. Merkert J, Butz S, Nieczaj R, Steinhagen-Thiessen E, Eckardt R. Combined whole body vibration and balance training using Vibrosphere: improvement of trunk stability, muscle tone, and postural control in stroke patients during early geriatric rehabilitation. Z Gerontol Geriatr. 2011;44(4):256-261. doi:10.1007/s00391-011-0170-9.
3. Brogårdh C, Flansbjer UB, Lexell J. No specific effect of whole-body vibration training in chronic stroke: a double-blind randomized controlled study. Arch Phys Med Rehabil. 2012;93(2):253-258. doi:10.1016/j.apmr.2011.09.005.
4. Lau RWK, Yip SP, Pang MYC. Whole-body vibration has no effect on neuromotor function and falls in chronic stroke. Med Sci Sports Exerc. 2012;44(8):1409-1418. doi:10.1249/MSS.0b013e31824e4f8c.
5. Marín PJ, Ferrero CM, Menéndez H, Martín J, Herrero AJ. Effects of whole-body vibration on muscle architecture, muscle strength, and balance in stroke patients: a randomized controlled trial. Am J Phys Med Rehabil. 2013;92(10):881-888. doi:10.1097/PHM.0b013e318292336c.
6. Nagino K, et al. Effect of whole-body vibration training on body balance in chronic stroke patients. Journal of Allied Health Sciences. 2013;4(2):24-31.

7. Lee G. Does whole-body vibration training in the horizontal direction have effects on motor function and balance of chronic stroke survivors? A preliminary study. J Phys Ther Sci. 2015;27(4):1133-1136. doi:10.1589/jpts.27.1133.
8. Liao LR, Ng GYF, Jones AYM, Huang MZ, Pang MYC. Whole-Body Vibration Intensities in Chronic Stroke: A Randomized Controlled Trial. Med Sci Sports Exerc. 2016;48(7):1227-1238. doi:10.1249/MSS.0000000000000909.
9. Lee JH, Kim SB, Lee KW, Lee SJ, Park H, Kim DW. The effect of a whole-body vibration therapy on the sitting balance of subacute stroke patients: a randomized controlled trial. Top Stroke Rehabil. 2017;24(6):457-462. doi:10.1080/10749357.2017.1305655.
10. Lee A, Kim H, Kim J, Choi DS, Jung JH, Lee J, et al. Modulating effects of whole-body vibration on cortical activity and gait function in chronic stroke patients. Brain Neurorehabil. 2020;13(2):e12. doi:10.12786/bn.2020.13.e12.
11. Sade I, Çekmece Ç, İnanır M, Selçuk B, Dursun N, Dursun E. The Effect of Whole Body Vibration Treatment on Balance and Gait in Patients with Stroke. Noro Psikiyatr Ars. 2020;57(4):308-311. doi:10.29399/npa.23380.
12. Kim JW, Lee JH. Effect of whole-body vibration therapy on lower extremity function in subacute stroke patients. J Exerc Rehabil. 2021;17(3):158-163. doi:10.12965/jer.2142246.123.
13. Xie L, Yi SX, Peng QF, Liu P, Jiang H. Retrospective study of effect of whole-body vibration training on balance and walking function in stroke patients. World J Clin Cases. 2021;9(22):6268-6277. doi:10.12998/wjcc.v9.i22.6268.
14. Wei N, Cai M. Optimal frequency of whole body vibration training for improving balance and physical performance in the older people with chronic stroke: A randomized controlled trial. Clin Rehabil. 2022;36(3):342-349. doi:10.1177/02692155211050564.
15. Xiao L, Liu C, Li Y, Deng Y, Xie B, Lin F, et al. Effects of whole body vibration combined with extracorporeal shock wave therapy on spasticity and balance gait parameters in hemiplegic patients with stroke. Zhong Nan Da Xue Xue Bao Yi Xue Ban. 2022;47(6):755-761. doi:10.11817/j.issn.1672-7347.2022.220063.
16. Majeed Y, Arshad H, Khattak HG, et al. Effects of plantar vibration on balance and postural instability in chronic stroke patients. Res Med Sci Rev. 2024;2:884-891.
17. Xu P, Song J, Fan W, et al. Impact of whole-body vibration training on ankle joint proprioception and balance in stroke patients: a prospective cohort study. BMC Musculoskelet Disord. 2024;25:768. doi:10.1186/s12891-024-07906-z.
18. Yoon H, Park C. Effectiveness of Proprioceptive Body Vibration Rehabilitation on Motor Function and Activities of Daily Living in Stroke Patients with Impaired Sensory Function. Healthcare. 2024;12(1):35. doi:10.3390/healthcare12010035.
19. Annino G, Alashram A, Romagnoli C, Iovane A, Youssef TM, Tancredi V, et al. Efficacy of localized muscle vibration on lower extremity functional ability in patients with stroke: A randomized controlled trial. J Bodyw Mov Ther. 2025;42:769-776. doi:10.1016/j.jbmt.2025.02.004.

Table S3. Detailed vibration-intervention characteristics of the included studies.

Study	Intervention & Control Protocol	Vibration Parameters and Schedule	Posture / Irradiation Site	Device Type
Annino et al., 2025 [28]	LMV + conventional physical therapy vs conventional physical therapy	30 Hz, 0.2 mm; 3 sessions/week; 8 weeks	Quadriceps tendon region	FOV1 Power Club
Brogårdh et al., 2012 [16]	WBV vs sham WBV	25 Hz, 3.75 mm; 2 sessions/week; 6 weeks	Standing, knees flexed 45–60°	Xrsize
Kim et al., 2021 [29]	WBV + squat training vs squat training	16 Hz; 5 sessions/week; 2 weeks	Upright standing with assisted squats	Sonix SW-VM10
Lau et al., 2012 [30]	WBV + exercise vs exercise	20–30 Hz, 0.44–0.60 mm; 3 sessions/week; 8 weeks	Standing	Jet-Vibe
Lee et al., 2015 [31]	Horizontal WBV + rehabilitation vs rehabilitation	1–3 Hz, 30 mm; 3 sessions/week; 6 weeks	Horizontal standing	Extream 1000
Lee et al., 2017 [32]	WBV + physical therapy vs physical therapy	40 Hz; 5 sessions/week; 2 weeks	Sitting posture	Sonix®
Lee et al., 2020 [33]	WBV vs sham WBV	20 Hz, 4 mm; single session	Half squat	Galileo Med S
Liao et al., 2016 [34]	Low-intensity WBV or High-intensity WBV + exercise vs exercise	20 or 30 Hz, 1 mm; 3 sessions/week; 4 weeks	Standing with squat and weight shift tasks	Gymna Fitvibe
Majeed et al., 2024 [35]	Plantar vibration + physical therapy vs physical therapy	20–40 Hz; 3 sessions/week; 6 weeks	Plantar surface of affected foot	OTO ER-839S
Marín et al., 2013 [36]	WBV + isometric rehabilitation vs isometric rehabilitation	5–21 Hz, 4–6 mm; 1–2 sessions/week; 12 weeks	Semi-squat standing	Galileo Home
Merkert et al., 2011 [37]	WBV + geriatric rehabilitation vs geriatric rehabilitation	35 Hz; 5 sessions/week; 3 weeks	Seated, supine, or standing	Vibrosphere
Nagino et al., 2013 [38]	WBV + rehabilitation vs rehabilitation	20 Hz, 3 mm; 3 sessions/week; 4 weeks	Squatting posture	Galileo 900
Sade et al., 2020 [39]	WBV + rehabilitation vs rehabilitation	35–40 Hz, 2 mm; daily; 3 weeks	Standing	Power Plate
van Nes et al., 2006 [15]	WBV + exercise vs exercise	30 Hz, 3 mm; 5 sessions/week; 6 weeks	Standing on Galileo platform	Galileo 900

Study	Intervention & Control Protocol	Vibration Parameters and Schedule	Posture / Irradiation Site	Device Type
Wei et al., 2022 [40]	WBV + physiotherapy vs physiotherapy	13 or 26 Hz, 3.4 mm; 5 sessions/week; 2 weeks	Standing barefoot, knees flexed 30°	Galileo Delta A
Xiao et al., 2022 [41]	WBV + ESWT + conventional treatment vs sham WBV + ESWT + conventional treatment	6–10 Hz, 4 mm; 5 sessions/week; 4 weeks	Standing with balance tasks	i-vib5050
Xie et al., 2021 [42]	WBV + routine rehabilitation vs routine rehabilitation	20 Hz, 5.2 mm; 6 sessions/week; 4 weeks	Standing, knees flexed 30–45°	Galileo Med L Plus
Xu et al., 2024 [43]	WBV + rehabilitation vs rehabilitation	10–20 Hz, 4–6 mm; 6 sessions/week; 4 weeks	Standing	Trunsan S110
Yoon et al., 2024 [44]	Proprioceptive body vibration + conventional physical therapy vs conventional physical therapy	20–30 Hz; 5 sessions/week; 8 weeks	Standing	Galileo

Note. Detailed intervention information was transferred from the main manuscript Table 1 to improve readability. WBV, whole-body vibration; LMV, local muscle vibration; ESWT, extracorporeal shock wave therapy.

Table S4. Residual heterogeneity following multilevel moderator analyses

Moderator	Studies	Effect sizes	F (df1, df2)	Raw p	FDR p	Residual τ²	Δτ² (%)
Duration	19	22	4.33 (2, 10.75)	0.042	0.293	0.623	-6.2
Frequency/week	19	22	0.55 (2, 3.15)	0.623	0.872	0.534	9.0
Frequency (Hz)	19	22	0.52 (1, 3.24)	0.521	0.872	0.568	3.2
Frequency pattern	19	22	0.01 (1, 14.95)	0.905	0.905	0.626	-6.7
Amplitude	14	17	2.65 (2, 5.33)	0.159	0.556	0.503	14.3
Vibration type	19	22	0.06 (1, 2.79)	0.822	0.905	0.624	-6.4
Disease stage	19	22	0.47 (1, 9.84)	0.508	0.872	0.591	-0.8

Note. Studies denotes the number of independent parent studies; effect sizes denotes the number of contributing effect-size estimates. Moderator analyses used three-level REML models with study-clustered CR2 inference. FDR-adjusted *p* values were obtained using the Benjamini–Hochberg procedure across the seven omnibus moderator tests. Residual τ^2 represents between-study variance after inclusion of the moderator. $\Delta\tau^2$ (%) was calculated relative to the primary between-study variance ($\tau^2 = 0.586$), with positive values indicating a reduction in residual between-study heterogeneity and negative values indicating an increase. Amplitude data were available for 14 studies contributing 17 effect-size estimates.

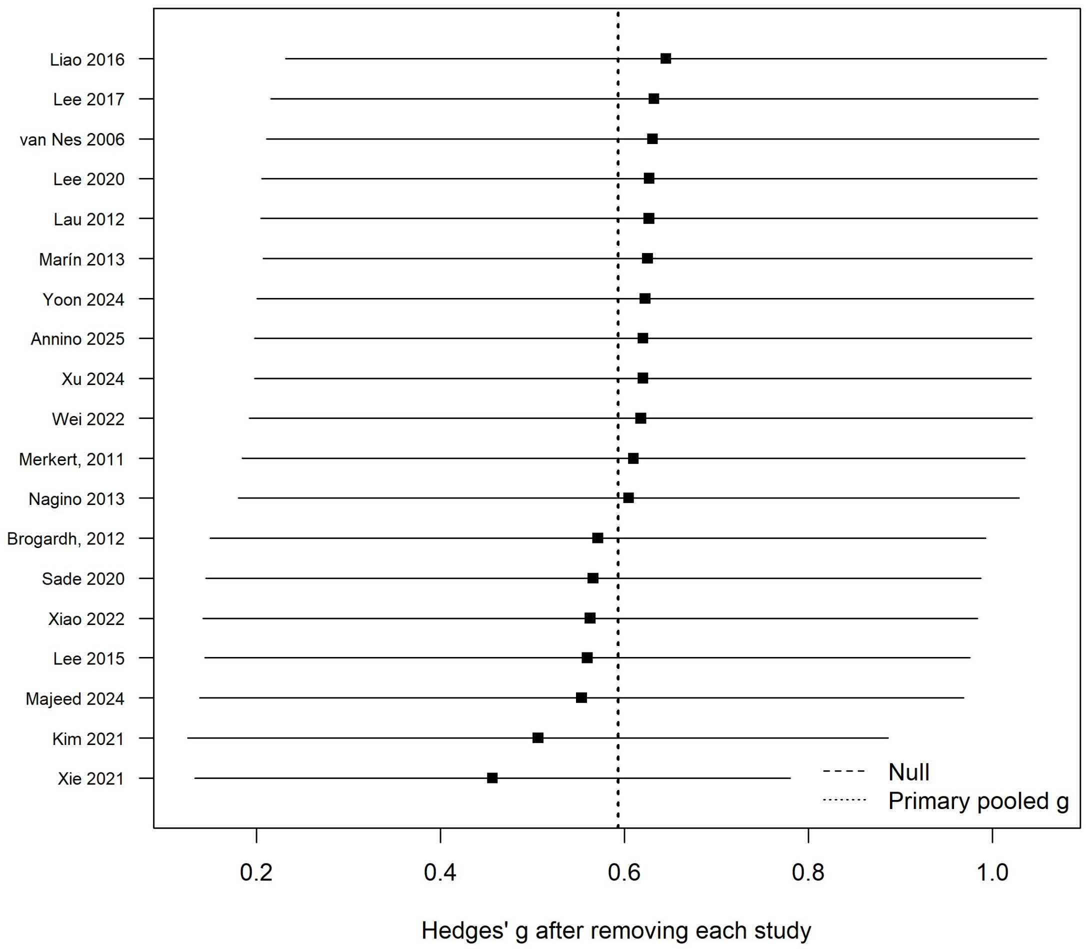

Figure S3. Leave-one-study-out analysis of the primary pooled effect.

Each point represents the pooled Hedges' g obtained after exclusion of one entire parent study, with horizontal lines indicating 95% confidence intervals. The vertical dotted line represents the primary pooled effect. Across the 19 study-deletion analyses, pooled estimates ranged from g = 0.457 to 0.645, and the pooled effect remained statistically significant in every analysis. Exclusion of Xie et al. produced the largest change in the pooled estimate.

Table S5. GRADE evidence profile of vibration therapy compared with control interventions for clinical balance outcomes after stroke.
Question: What is the certainty of evidence for vibration therapy compared with control interventions for clinical balance outcomes after stroke?

Certainty assessment							№ of patients		Effect		Certainty	Importance
№ of studies	Study design	Risk of bias	Inconsistency	Indirectness	Imprecision	Other considerations	vibration therapy	control interventions	Relative (95% CI)	Absolute (95% CI)		
**RCTs**												
16	randomised trials	serious[a]	serious[b]	not serious	not serious[c]	none[d]	406	383	-	0.507 (0.141 to 0.873)	⊕⊕◯◯ Low[a,b]	CRITICAL
**Non-RCTs**												
3	non-randomised studies	very serious[e]	very serious[f]	not serious	serious[g]	Not assessed separately[h]	104	105	-	0.937 (-0.697 to 2.571)	⊕◯◯◯ Very low[e,f,g]	CRITICAL

CI: confidence interval

a. Risk of bias (RCTs): Downgraded one level for serious risk of bias. Eight of the 16 randomized controlled trials (50.0%) were judged to be at high overall risk of bias. Moreover, exclusion of studies judged to be at high or serious risk of bias attenuated the pooled effect to Hedges' $g = 0.161$ (95% CI −0.116 to 0.439; $p = 0.217$), indicating sensitivity of the pooled effect to methodological quality.

b. Inconsistency (RCTs): Downgraded one level for serious inconsistency. Significant residual heterogeneity remained in the RCT-only analysis (QE(18) = 65.85, $p < 0.001$; between-study $\tau^2 = 0.363$), and the 95% prediction interval ranged from −0.831 to 1.845, indicating substantial variability in treatment effects across settings.

c. Imprecision (RCTs): Not downgraded. The estimate was based on 16 independent randomized trials, and the 95% confidence interval (0.141 to 0.873) excluded the null. The prediction interval was considered under inconsistency rather than being used to duplicate downgrading for imprecision.

d. Publication bias (RCTs): Not downgraded. Small-study-effect diagnostics for the overall evidence were inconsistent: the Egger-type and weighted regression tests were not statistically significant (both $p = 0.397$), whereas the Begg–Mazumdar test was significant (Kendall's $\tau = 0.359$, $p = 0.019$). Trim-and-fill estimated no missing studies. These diagnostics were interpreted cautiously because they did not fully account for within-study dependence.

e. Risk of bias (non-RCTs): Downgraded for very serious risk of bias. Two of the three non-randomized studies (66.7%) were judged to be at serious overall risk of bias, with major concerns related primarily to confounding and participant selection.

f. Inconsistency (non-RCTs): Downgraded for very serious inconsistency. Heterogeneity among the three non-randomized studies was extreme ($Q(2) = 57.56$, $p < 0.001$; $\tau^2 = 1.998$), and the 95% prediction interval was very wide (−2.279 to 4.154), indicating marked variation in treatment effects.

g. Imprecision (non-RCTs): Downgraded for serious imprecision. Only three independent non-randomized studies contributed to the estimate, and the pooled 95% confidence interval was wide and crossed the null (Hedges' $g = 0.937$, 95% CI −0.697 to 2.571; $p = 0.261$).

h. Publication bias (non-RCTs): Publication bias was not assessed separately because only three non-randomized studies were available, which was insufficient for meaningful funnel-plot asymmetry testing.

Table S6. Effect-size-level data contributing to the primary meta-analysis

Study	Outcome	nI	nC	Change I	SD change I	Change C	SD change C	Hedges' g	vi	Comparison	Time point
Annino 2025	BBS	19	18	2.18	12.824	0.94	8.191	0.112	0.10836	LMV + CPT vs CPT	Immediate post-treatment
Brogårdh 2012	BBS	16	15	2.1	2.498	−0.30	2.042	1.021	0.14598	WBV vs sham WBV	Immediate post-treatment
Kim 2021	BBS	20	18	11.4	2.88	4	3.46	2.287	0.1744	WBV + squat training vs squat training	Immediate post-treatment
Lau 2012	BBS	41	41	1.5	7.535	1.2	5.103	0.046	0.04879	WBV + exercise vs exercise	Immediate post-treatment
Lee 2015	BBS	12	9	6	5.17	0.56	0.88	1.314	0.23553	Horizontal WBV + rehabilitation vs rehabilitation	Immediate post-treatment
Lee 2017	BBS	15	15	2.1	2.2	2.6	4.5	−0.137	0.13365	WBV + physical therapy vs physical therapy	Immediate post-treatment
Lee 2020	TPOMA-B	15	14	0.07	3.325	0	3.15	0.021	0.1381	WBV vs sham WBV	Immediate post-treatment
Lee 2020	TPOMA-T	15	14	0.14	5.202	0	5.21	0.026	0.13811	WBV vs sham WBV	Immediate post-treatment
Liao 2016a	Mini-BESTest	28	28	4.9	4.912	6.1	4.431	−0.253	0.072	Low-intensity WBV + exercise vs exercise	Immediate post-treatment
Liao 2016b	Mini-BESTest	28	28	5	4.651	6.1	4.431	−0.239	0.07194	High-intensity WBV + exercise vs exercise	Immediate post-treatment
Majeed 2024	BBS	16	16	9.69	4.415	4	3.733	1.357	0.15376	Plantar vibration + physical therapy vs physical therapy	Immediate post-treatment
Marín 2013	BBS	11	9	2.3	4.703	2.8	10.978	−0.059	0.20211	WBV + isometric rehabilitation vs isometric rehabilitation	Immediate post-treatment
Merkert 2011	BBS	25	23	12.2	10.7	9.1	8.3	0.317	0.08452	WBV + geriatric rehabilitation vs geriatric rehabilitation	Immediate post-treatment

Nagino 2013	BBS	15	15	4	8.663	0	11.053	0.392	0.13589	WBV + rehabilitation vs rehabilitation	Immediate post-treatment
Sade 2020	BBS	26	17	11.2	5.057	2.4	10.95	1.094	0.11119	WBV + rehabilitation vs rehabilitation	Immediate post-treatment
van Nes 2006	BBS	27	26	16.7	13.908	17.4	16.866	−0.045	0.07552	WBV + exercise vs exercise	Immediate post-treatment
Wei 2022a	BBS	26	26	−0.24	3.81	−0.77	3.46	0.143	0.07712	13-Hz WBV + physiotherapy vs physiotherapy	Immediate post-treatment
Wei 2022b	BBS	26	26	0.27	4.26	−0.77	3.46	0.264	0.07759	26-Hz WBV + physiotherapy vs physiotherapy	Immediate post-treatment
Xiao 2022	BBS	25	25	17.09	5.286	11.78	3.813	1.134	0.09286	WBV + ESWT + conventional treatment vs sham WBV + ESWT + conventional treatment	Immediate post-treatment
Xie 2021	BBS	65	65	18.32	2.18	13.29	1.66	2.581	0.05639	WBV + routine rehabilitation vs routine rehabilitation	Immediate post-treatment
Xu 2024	BBS	17	18	10.744	9.924	9.589	10.377	0.111	0.11456	WBV + rehabilitation vs rehabilitation	Immediate post-treatment
Yoon 2024	BBS	22	22	17.05	14.885	15.91	11.455	0.084	0.09099	Proprioceptive body vibration + CPT vs CPT	Immediate post-treatment

Note. Effect sizes represent standardized between-group differences in change from baseline. Directly reported change means and standard deviations were used when available. When change means were not reported, they were calculated as post-intervention minus baseline values. When change-score standard deviations were unavailable, they were reconstructed from pre- and post-intervention standard deviations using an assumed within-group pre-post correlation of r = 0.50. Hedges' small-sample correction was applied to all standardized mean differences. Positive Hedges' g values favor vibration therapy. Multiple eligible intervention arms or outcomes from the same parent study were retained as separate effect-size estimates and accounted for in the dependency-aware analyses. nI, intervention-

group sample size; nC, control-group sample size; BBS, Berg Balance Scale; TPOMA-B, Tinetti Performance-Oriented Mobility Assessment balance score; TPOMA-T, Tinetti Performance-Oriented Mobility Assessment total score; Mini-BESTest, Mini-Balance Evaluation Systems Test; vi, sampling variance. The Comparison column identifies the intervention and comparator contributing to each estimate, and the Time point column identifies the assessment used in the primary synthesis; all primary estimates used the immediate post-treatment assessment.

Table S7. Sensitivity and robustness analyses of the pooled effect of vibration therapy on clinical balance outcomes

Analysis	Studies	Effect sizes	Hedges' g	95% CI	p	Between-study τ²	95% prediction interval
Primary analysis	19	22	0.593	0.191 to 0.995	0.006	0.586	-1.066 to 2.252
Pre-post r = .25	19	22	0.549	0.160 to 0.938	0.008	0.545	-1.051 to 2.148
Pre-post r = .75	19	22	0.686	0.248 to 1.124	0.004	0.714	-1.142 to 2.515
Within-study ρ = .30	19	22	0.595	0.193 to 0.998	0.006	0.586	-1.063 to 2.254
Within-study ρ = .50	19	22	0.597	0.194 to 0.999	0.006	0.586	-1.061 to 2.255
Within-study ρ = .70	19	22	0.598	0.195 to 1.001	0.006	0.585	-1.059 to 2.256
RCT-only	16	19	0.507	0.141 to 0.873	0.01	0.363	-0.831 to 1.845
High/serious RoB excluded	10	12	0.161	-0.116 to 0.439	0.217	0.057	-0.459 to 0.781
TPOMA-total excluded	19	21	0.596	0.193 to 0.999	0.006	0.587	-1.064 to 2.255
BBS-only	17	18	0.684	0.251 to 1.118	0.004	0.596	-1.009 to 2.378
Baseline-imbalance study excluded	18	21	0.571	0.149 to 0.993	0.011	0.614	-1.135 to 2.277
Primary post-treatment time point	19	22	0.593	0.191 to 0.995	0.006	0.586	-1.066 to 2.252
Outlier study excluded (Xie 2021)	18	21	0.457	0.133 to 0.780	0.009	0.317	-0.776 to 1.689

Note. All analyses used standardized between-group differences in change from baseline expressed as Hedges' *g*. The primary analysis assumed a within-group pre-post correlation of $r = 0.50$ when change-score standard deviations required reconstruction. Alternative pre-post correlations of $r = 0.25$ and 0.75 were examined as effect-size assumption sensitivity analyses. Sensitivity analyses assuming within-study correlations (ρ) of 0.30, 0.50, and 0.70 were additionally performed to examine plausible dependence among multiple estimates from the same parent study. Unless otherwise specified, statistical inference was based on multilevel REML models with study-clustered CR2 robust variance estimation. Studies denotes the number of independent parent studies; effect sizes denotes the number of contributing effect-size estimates. RoB, risk of bias; BBS, Berg Balance Scale; TPOMA, Tinetti Performance-Oriented Mobility Assessment; CI, confidence interval.

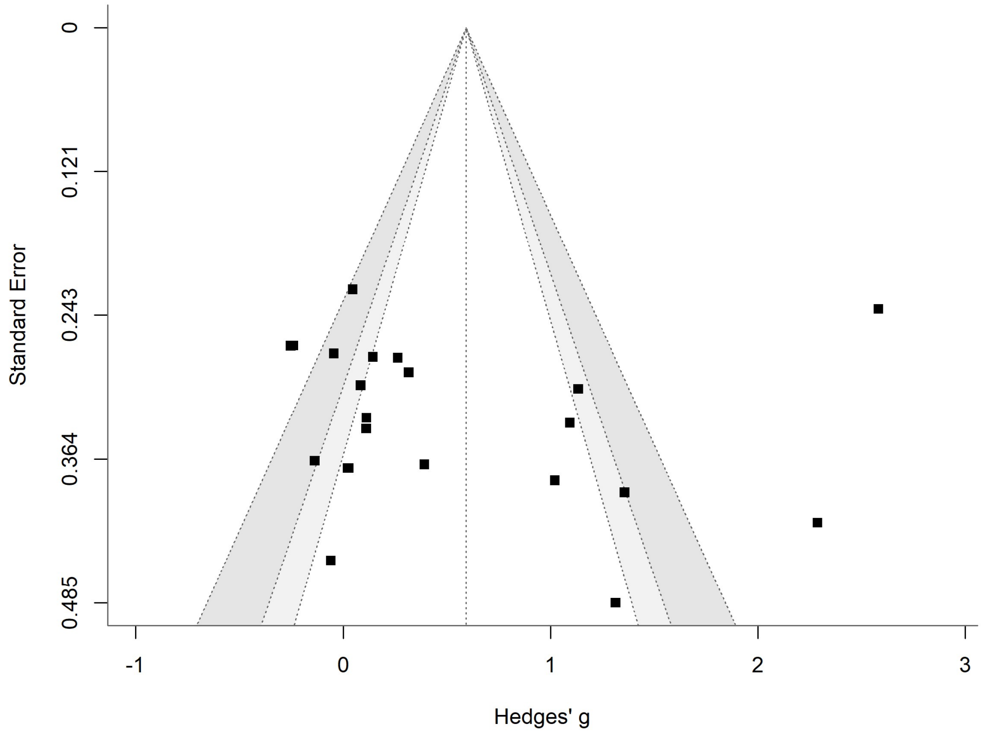

Figure S4. Funnel plot of the effect-size estimates contributing to the primary meta-analysis.
The plot displays Hedges' g against its standard error for 22 effect-size estimates. The Egger-type and weighted regression tests were not statistically significant (both p = 0.397), whereas the Begg–Mazumdar rank-correlation test was significant (Kendall's $\tau = 0.359$, $p = 0.019$). Trim-and-fill estimated no missing studies. Because the conventional diagnostics were conducted at the effect-size level, they do not fully account for within-study dependence and were interpreted as exploratory.

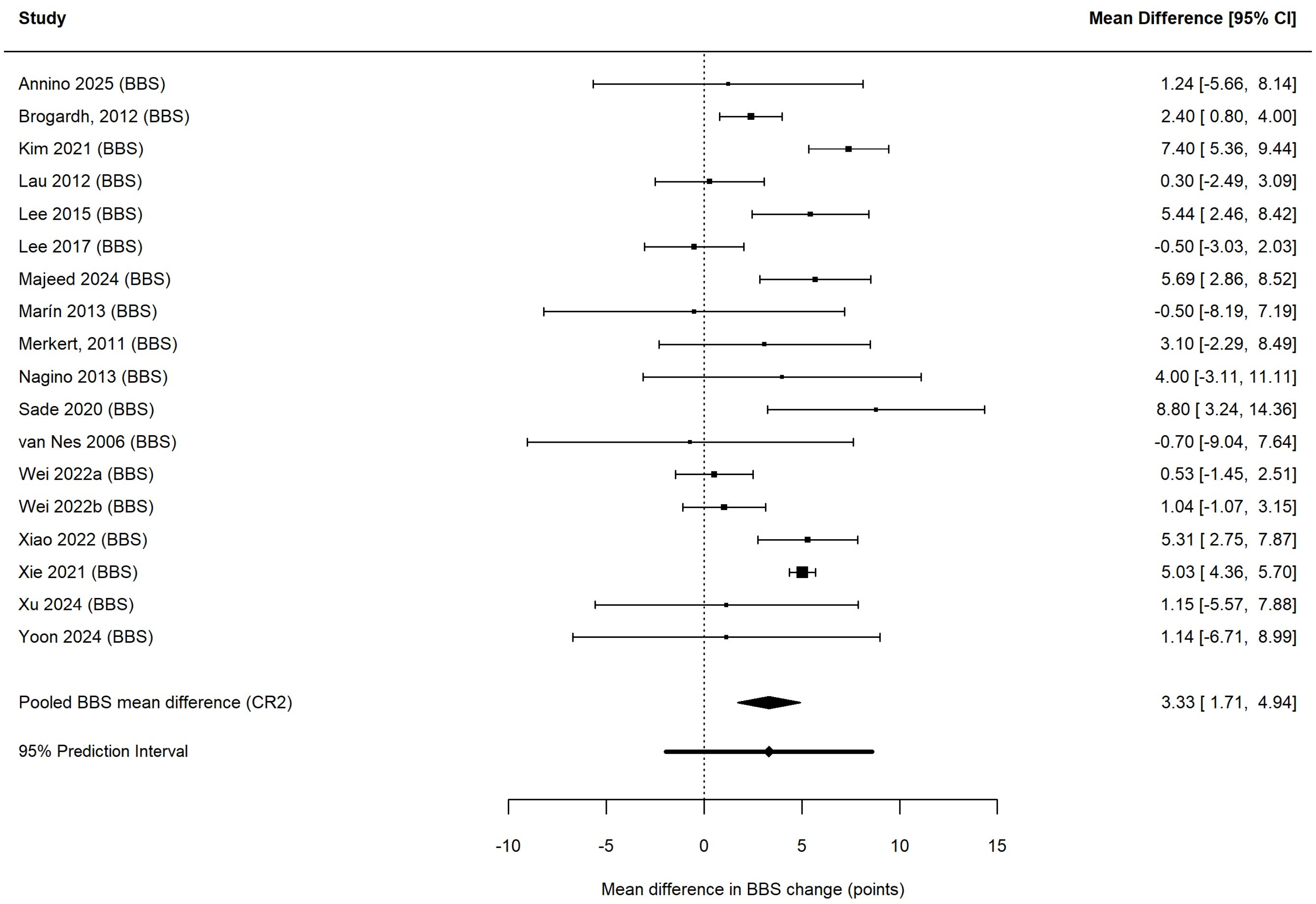

Figure S5. Forest plot of the BBS raw mean-difference analysis.

Seventeen studies contributed 18 BBS effect-size estimates. The pooled between-group mean difference in BBS change favored vibration therapy by 3.326 points (95% CI 1.714 to 4.938, $p < 0.001$) using a multilevel REML model with study-clustered CR2 inference. The 95% prediction interval ranged from -1.952 to 8.604 BBS points.
